# Detection of Mitotic Neuroblasts Provides Additional Evidence of Steady-State Neurogenesis in the Adult Small Intestinal Myenteric Plexus

**DOI:** 10.1523/ENEURO.0005-24.2025

**Published:** 2025-03-05

**Authors:** Anastazja M. Gorecki, Jared Slosberg, Su Min Hong, Philippa Seika, Srinivas N. Puttapaka, Blake Migden, Anton Gulko, Alpana Singh, Chengxiu Zhang, Rohin Gurumurthy, Subhash Kulkarni

**Affiliations:** ^1^Perron Institute for Neurological and Translational Science, Nedlands, Western Australia 6009, Australia; ^2^School of Biological Sciences, University of Western Australia, Crawley, Western Australia 6009, Australia; ^3^Department of Genetic Medicine, Johns Hopkins University – School of Medicine, Baltimore, Maryland 21205; ^4^Division of Gastroenterology, Department of Medicine, Beth Israel Deaconess Medical Center, Boston, Massachusetts 02115; ^5^Division of Endocrinology, Department of Medicine, Beth Israel Deaconess Medical Center, Boston, Massachusetts 02115; ^6^Center for Neurogastroenterology, Department of Medicine, Johns Hopkins University–School of Medicine, Baltimore, Maryland 21205; ^7^Division of Medical Sciences, Harvard Medical School, Boston, Massachusetts 02115; ^8^Program in Neurosciences, Harvard Medical School, Boston, Massachusetts 02115

**Keywords:** adult neurogenesis, DNA content, enteric nervous system, flow analyses, neuroblasts, phosphor-histone H3

## Abstract

Maintenance of normal structure of the enteric nervous system (ENS), which regulates key gastrointestinal functions, requires robust homeostatic mechanisms, since by virtue of its location within the gut wall, the ENS is subject to constant mechanical, chemical, and biological stressors. Using transgenic and thymidine analog-based experiments, we previously discovered that neuronal turnover—where continual neurogenesis offsets ongoing neuronal loss at steady state—represents one such mechanism. Although other studies confirmed that neuronal death continues into adulthood in the myenteric plexus of the ENS, the complicated nature of thymidine analog presents challenges in substantiating the occurrence of adult neurogenesis. Therefore, it is vital to employ alternative, well-recognized techniques to substantiate the existence of adult enteric neurogenesis in the healthy gut. Here, by using established methods of assessing nuclear DNA content and detecting known mitotic marker phosphor-histone H3 (pH3) in Hu^+^ adult ENS cells, we show that ∼10% of adult small intestinal myenteric Hu^+^ cells in mice and ∼20% of adult human small intestinal myenteric Hu^+^ cells show evidence of mitosis and hence are cycling neuroblasts. We observe that proportions of Hu^+^ cycling neuroblasts in the adult murine ENS neither vary with ganglionic size nor do they differ significantly between two intestinal regions, duodenum and ileum, or between sexes. Confocal microscopy provides further evidence of cytokinesis in Hu^+^ cells. The presence of a significant population of cycling neuroblasts in adult ENS provides further evidence of steady-state neurogenesis in the adult ENS.

## Significance Statement

Using three-dimensional confocal microscopy, immunohistochemical detection of cell cycle marker phosphor-histone H3, and DNA content assessments using flow cytometry in Hu^+^ cells from adult small intestinal murine myenteric plexus, we show that ∼10% of Hu^+^ cells in adult gut are mitotic neuroblasts, whose proportional representation does not significantly differ between sexes or small intestinal regions. We further test and observe mitotic marker pH3 also immunolabels ∼23% of adult human myenteric Hu^+^ cells suggesting that the presence of mitotic neuroblasts also extends to the adult human gut. These data further evidence the steady-state adult enteric neurogenesis in the healthy gut and provide important cellular details in understanding how precursor cells continually generate large numbers of adult neurons in the healthy gut.

## Introduction

The cells of the enteric nervous system (ENS) are located entirely within the gut wall and are routinely exposed to significant and continual mechanical, chemical, and biological insults (Gregersen and Kassab, 1996; Sherman et al., 2015; Hyland and Cryan, 2016). Despite these ongoing and significant stressors, the question of how the adult ENS maintains itself has not been adequately addressed, especially given prior reports from multiple groups that a significant proportion of enteric neurons undergo apoptosis at steady state (Nezami et al., 2014; Kozlowska et al., 2016, 2018).

In a previous study, we reported the presence of a neurogenic mechanism, which gives rise to a significant number of new neurons at steady state in the adult gut (Kulkarni et al., 2017). Using thymidine analog studies and multiple lines of evidence to test for neuronal apoptosis, we demonstrated the existence of an adult enteric neurogenic program in the adult murine myenteric plexus (MP) that generates new neurons to replace dying apoptotic neurons, which account for ∼10% of all myenteric neurons at any given time. We proposed that this is one mechanism to maintain the structural and functional integrity of the adult ENS, especially in the MP tissue. While other studies provide independent validation of the high rate of neuronal apoptosis in the adult ENS (Chandrasekharan et al., 2011; Nezami et al., 2014; Becker et al., 2018; De Schepper et al., 2018; Ye et al., 2020), the complicated nature of thymidine analog-based assays and their susceptibility to variations in tissue handling and fixation have made it difficult for investigators to consistently detect neurogenesis (Kulkarni and Pasricha, 2022). Thus, there is a need to use alternate methods that can aid in the detection of adult neurogenesis and provide further evidence of a high rate of neurogenesis that counteracts the equally high rate of neuronal apoptosis.

In addition, we also recently showed the presence of significant heterogeneity in myenteric ganglia (Kobayashi et al., 2021; Hamnett et al., 2022). Myenteric ganglia exist in various sizes, as defined by their neuronal numbers, and in the small intestine can contain from 3 to 150 neurons (Kobayashi et al., 2021). The relative distribution of the ganglionic size in the intestine was skewed significantly toward smaller ganglia (i.e., containing fewer neurons; Kobayashi et al., 2021). Similarly, proportions of various neuronal subpopulations contained within these ganglia show significant differences across regions of the intestine (Hamnett et al., 2022). This heterogeneity begs the question of whether the rates of neurogenesis and neuronal loss are equally represented in the diverse ganglia of MP in differing locations in the small intestine and across the two sexes. Since thymidine analog-based assays, which detect the presence of these chemicals dosed to animals over several days, do not permit the study of ongoing neurogenesis at a defined snapshot of time, these assays are unsuitable for providing a real-time assessment of putative differences in neurogenic niches across ganglia and locations. Furthermore, despite high rates of apoptosis (which range from 5 to 30% of all myenteric neurons) reported in human ENS (Chandrasekharan et al., 2011; Kozlowska et al., 2016, 2018), the presence of a neurogenic mechanism in the human ENS is unclear. Since thymidine analogs cannot be administered to human beings, these assays cannot be translated to human tissues, and thus, there is a need to simplify the protocols to facilitate better assessment of neurogenesis both in mice and in human specimens.

To address these issues, in this report, we use three different methods to interrogate whether neurogenesis occurs in adult healthy small intestinal ENS. First, using high-resolution confocal microscopy, we found evidence of binucleated Hu-immunolabeled ganglionic cells in the adult small intestinal MP. Second, by performing a flow cytometry-based estimation of DNA content in myenteric Hu-immunolabeled nuclei, we observe DNA content suggestive of S-phase and G2/M phase in significant proportions of Hu-immunolabeled nuclei—which are indicative of active DNA synthesis and mitosis in a population of adult murine myenteric neurons. Finally, by performing immunostaining with an established mitotic marker phosphor-histone H3 (pH3), we identified the presence of mitotic Hu-immunolabeled cells in the adult murine and human ENS. The proportions of pH3-immunoreactive neurons in the adult murine MP were found to match the previously observed proportions for apoptotic neurons and were conserved between myenteric ganglia regardless of their size, location in the small intestine, and the sex of the animals. Thus, these three different methods together provide additional evidence of continuous neurogenesis in the adult healthy gut.

## Materials and Methods

### Animals

Experimental protocols were approved by Johns Hopkins University (JHU) Animal Care and Use Committee and the Animal Care and Use Committee at Beth Israel Deaconess Medical Center (BIDMC) in accordance with the guidelines provided by the National Institutes of Health. Age-matched 2–3-month-old adult wild-type C57BL/6 mice were used. Both male and female mice were used for the pH3-immunostaining experiments, and male mice were used for all the other experiments.

### Isolation and immunolabeling of neuronal nuclei from the adult longitudinal muscle–MP (LM–MP) tissue

Mice were anesthetized with isoflurane and killed by cervical dislocation. For small intestinal tissue isolation, a laparotomy was performed, and the entire small intestine was removed and lavaged with PBS containing penicillin–streptomycin (Pen–Strep; Invitrogen). The small intestine was then cut into 1-cm-long segments. Next, tissues were placed over a sterile plastic rod, and a superficial longitudinal incision was made along the serosal surface, and the LM–MP was peeled off from the underlying tissue using a wet sterile cotton swab (Kulkarni et al., 2017) and directly frozen in liquid nitrogen and stored at −80°C for further processing. Two different protocols were used henceforth, which are detailed as follows.

BIDMC protocol: The frozen tissue was next transferred to a gentleMACs C Tube (Miltenyi Biotech) containing 2 ml of TST buffer (containing 60 μl of 5 M NaCl, 42 μl of 1 M MgCl_2_, 20 μl of 1 M Tris–HCl, 2 μl of 1 M CaCl_2_, 10 μl 2% bovine serum albumin (BSA), 6 μl 10% Tween 20, in 1,860 μl ultrapure water), pH 7.5, and allowed to thaw for 2 min. Thawed tissues were chopped using dissecting scissors for 45 s and then added to the C tube, which is installed on the gentleMACs dissociator (Miltenyi Biotech). The tissue was processed through two rounds of 268 rotations, for a total of 72 s dissociation time after which the tube was placed and incubated on ice for 10 min. Next, using a wide-bore, low-retention 1,000 μl pipette tip (Olympus, catalog #23-165RL), the suspension was transferred to a 40 μm prewet cell sieve (Thermo Fisher Scientific, catalog #22-363-547) that was set on top of a sterile 50 ml falcon tube and filtered through. The C tube was washed with 1 ml of 1× ST buffer (TST buffer minus BSA and Tween 20), and the buffer was next transferred to the cell sieve. The cell sieve was then washed with 2 ml of 1× ST buffer and then discarded. Using again the wide-bore, low-retention 1,000 μl pipette tip (Olympus, catalog #23-165RL), the filtered nuclei suspension was transferred to a prewet 40 μm cell sieve (Thermo Fisher Scientific, catalog #22-363-547) that is set on top of a 5 ml FACS tube. The suspension was spun down in a swing bucket centrifuge at 500 × *g* for 5 min and at 4°C. Centrifuge brake was set at half to ensure that the pellet is not disturbed. The top ∼4.9 ml of the supernatant was removed, and the pellet was washed with 5 ml PBS solution containing 0.02% BSA, which was added to the tube gently, and the tube was again spun at 500 × *g* for 5 min and at 4°C. The supernatant was removed, and the nuclear pellet was resuspended in 500 μl of PBS containing 0.02% BSA. The suspension was then split equally into two tubes. The directly conjugated antibody anti-Hu 488 (1:750, Abcam #ab237234; RRID:AB_3668757) was added to the first tube, while the second tube served as the control. A drop of NucBlue (Thermo Fisher Scientific) was added to both the tubes. Both the tubes were incubated on ice for 45 min, after which the nuclear suspensions were spun down at 500 × *g* for 5 min and at 4°C, with the centrifuge brakes again set to half. A 450 μl of supernatant from each tube was removed, and the nuclear pellet was resuspended in 450 μl of PBS solution containing 0.02% BSA. The suspensions were then analyzed for NucBlue fluorescence intensity (as a marker for DNA content) in Hu-immunofluorescent nuclei using the CytoFLEX flow cytometer.

Johns Hopkins protocol: The frozen and chopped tissue was placed into an ice-cold gentleMACS C tube with 3 ml of TST buffer. The tissue was dissociated on a gentleMACS Octo Dissociator using the *4C_nuclei_01* protocol with prechilled OctoCooler. Disrupted cell suspension was subsequently incubated on ice for 5 min before being filtered through a 40 μm prewet (with 1× ST buffer) cell sieve that is set on top of a 50 ml falcon tube. The C tube was rinsed with 1 ml ST buffer, and an additional 2 ml of 1× ST buffer was used to rinse the filter. The nuclei suspension was centrifuged in a swing bucket centrifuge at 500 × *g* for 5 min and at 4°C with the centrifuge brakes set to half. The pellet was resuspended in 5 ml PBS containing 1% BSA, centrifuged and pelleted again, and finally resuspended in PBS containing 1% BSA and 5% normal goat serum (NGS). This suspension was incubated on ice for 5 min after which the suspension is divided equally into two tubes. Anti-Hu 647 directly conjugated antibody (Abcam, ab237235; RRID:AB_3668731) was added at a concentration of 1:250 to the first but not the second tube, and both the tubes are incubated on ice for 45 min. Excess antibody was removed by washing the suspension twice (again with centrifugation at 500 × *g* for 5 min and at 4°C with the centrifuge brakes set to half), and the nuclei suspensions were finally suspended in 1 ml sterile PBS in a 15 ml conical tube. Next, a fixative was freshly prepared by adding 50 μl of 50 mg/ml dithiobis(succinimidyl propionate) in dimethyl sulfoxide solution to 4 ml of ice-cold methanol, dropwise while vortexing at moderate speed. The nuclei suspensions were fixed by adding the freshly prepared fixative dropwise to the nuclei while swirling the suspension to reduce clumping. The fixed nuclei were next incubated at 4°C for 10 min on an end-over-end rotor (speed of 15 rpm). Fixed nuclei were rehydrated by adding two volumes of PBS containing 0.1% Triton X-100 and then washed twice with PBS containing 1% BSA. DAPI was added to both tubes at a concentration of 1 μg/ml, and samples were filtered through a 40 μm before analyses on a flow analyzer (BD FACSAria III).

Flow data was analyzed on FlowJo 10.

### Tissue preparation

Mice were anesthetized with isoflurane and killed by cervical dislocation. For small intestinal tissue isolation, a laparotomy was performed, and the entire small intestine was removed and lavaged with PBS containing Pen–Strep (Invitrogen) and then cut into 2-cm-long segments, such that segments from the duodenum, jejunum, and ileum were separated. Next, tissues were placed over a sterile plastic rod, and a superficial longitudinal incision was made along the serosal surface and LM–MP was peeled off from the underlying tissue using a wet sterile cotton swab (Kulkarni et al., 2017) and placed in Opti-MEM medium (Invitrogen) containing Pen–Strep. The tissue was then laid flat and fixed with freshly prepared ice-cold 4% paraformaldehyde (PFA) solution for 4–5 min in the dark. For a small subset of tissues, where we tested how fixation alters immunoreactivity, we fixed LM–MP tissues for 10, 15, and 20 min with freshly prepared ice-cold PFA solution. All LM–MP tissues were fixed within 30 min of euthanasia. After the fixation, the tissue was stored in ice-cold sterile PBS with Pen–Strep for immunofluorescence staining and subsequent microscopy.

### Immunochemistry

For murine tissue: The fixed LM–MP tissue was washed twice in ice-cold PBS in the dark at 16°C. The tissue was incubated in blocking-permeabilizing buffer (BPB; 5% NGS with 0.3% Triton X-100) for 1 h, followed by incubation with either the combination of (1) anti-pH3 antibody (unconjugated antibody, EMD Millipore, #06-570; RRID:AB_310177, 1:500; conjugated antibody, #3458S, Cell Signaling Technology; 1:250; RRID:AB_10694086) and ANNA-1 (1:1,000; patient antisera against the neuronal Hu antigens; RRID:AB_2813895); (2) anti-cleaved caspase 3 (CC3; #Asp175, Cell Signaling Technology, 1:250; RRID:AB_2070042) and ANNA-1 (1:1,000); (3) anti-PGP9.5 (#ab108986, Abcam, 1:250; RRID:AB_10891773) and ANNA-1 (1:1,000); (4) anti-Hu (#ab184267, Abcam, 1:1,000; RRID:AB_2864321) and ANNA-1 (1:1,000); and (4) anti-Nestin (#NB100-1604, Novus; 1:250; RRID:AB_2282642) and anti-Hu (#ab184267, Abcam, 1:1,000) for 48 h at 16°C in the dark with shaking at 55 rpm. The tissues were then washed three times (15 min wash each) in PBS at room temperature in the dark and incubated in the appropriate secondary antibody (anti-human 488; RRID:AB_2536548; anti-chicken 488; RRID:AB_142924; and anti-rabbit 647; RRID:AB_2535813, Invitrogen, all at 1:500) at room temperature for 1 h while on a rotary shaker (65 rpm). The tissues were again washed three times in PBS at room temperature, counterstained with DAPI to stain the nuclei, overlaid with ProLong Antifade Gold mounting medium, coverslipped, and imaged. ANNA1 patient antiserum was a kind gift from Dr. Sean Pittock at Mayo Clinic.

For formalin fixed paraffin embedded (FFPE) human tissue: The use of the commercially procured human tissue was approved by the Institutional Review Board of BIDMC. FFPE tissue sections from human full-thickness duodenal tissue of four different patients (two males and two females) with no known GI dysmotility disorder were procured from a commercial vendor and from pathology archive BIDMC. To process them, slides were first baked at 55°C for 15 min, after which, they were deparaffinized and rehydrated by immersion through the following solutions: (1) two washes (5 min each) with xylene; (2) two washes with 100% ethanol (5 min each); (3) two washes with 95% ethanol (5 min each); (4) two washes with 70% ethanol (5 min each); and finally (5) two washes with deionized water (5 min each). Next, using sodium citrate buffer, pH 6.0, 40 min antigen retrieval was performed in a pressure cooker. The slides were then cooled in ice-cold 1× PBS. Slides were marked with a hydrophobic pen around the sections and then blocked and permeabilized in BPB for 1 h. Buffer was washed off with 1× PBS, and sections were incubated with either the combination of directly conjugated anti-pH3 antibody (EMD Millipore, 1:250) and directly conjugated anti-Hu antibody (1:300), or the combination of anti-CC3 (1:250) and ANNA-1 (1:500) for 24 h at 16°C in the dark. For tissue sections treated with anti-CC3 and anti-ANNA1 antibodies, following incubation with primary antibody, the sections were washed three times for 15 min each in PBS at room temperature in the dark and then incubated with the appropriate secondary antibodies (Invitrogen anti-human 488 and anti-rabbit 647; both at 1:500) at room temperature for 1 h. Next, the sections were again washed three times in PBS at room temperature, counterstained with DAPI to stain the nuclei, overlaid with ProLong Antifade Gold mounting medium, coverslipped, and imaged.

### Microscopy

Microscopy was performed using the oil immersion 40× objective on the Olympus Fluoview 3000rs confocal microscope with galvano and resonance scanning mode, with 40× water immersion objective on the Zeiss LSM880, and with 20× and 40× magnification of the EVOS M7000 microscope. Control tissues that were immunostained with secondary antibodies only were used to set the threshold laser intensities. In mice, all ANNA-1/Hu-immunostained neurons were included in the analysis. Image analyses were performed by using Fiji (NIH) or ImarisViewer (10.2.0).

## Results

### Hu-expression marks a subset of multinucleated ganglionic cells in adult small intestinal murine myenteric ganglia

**Figure 1.**
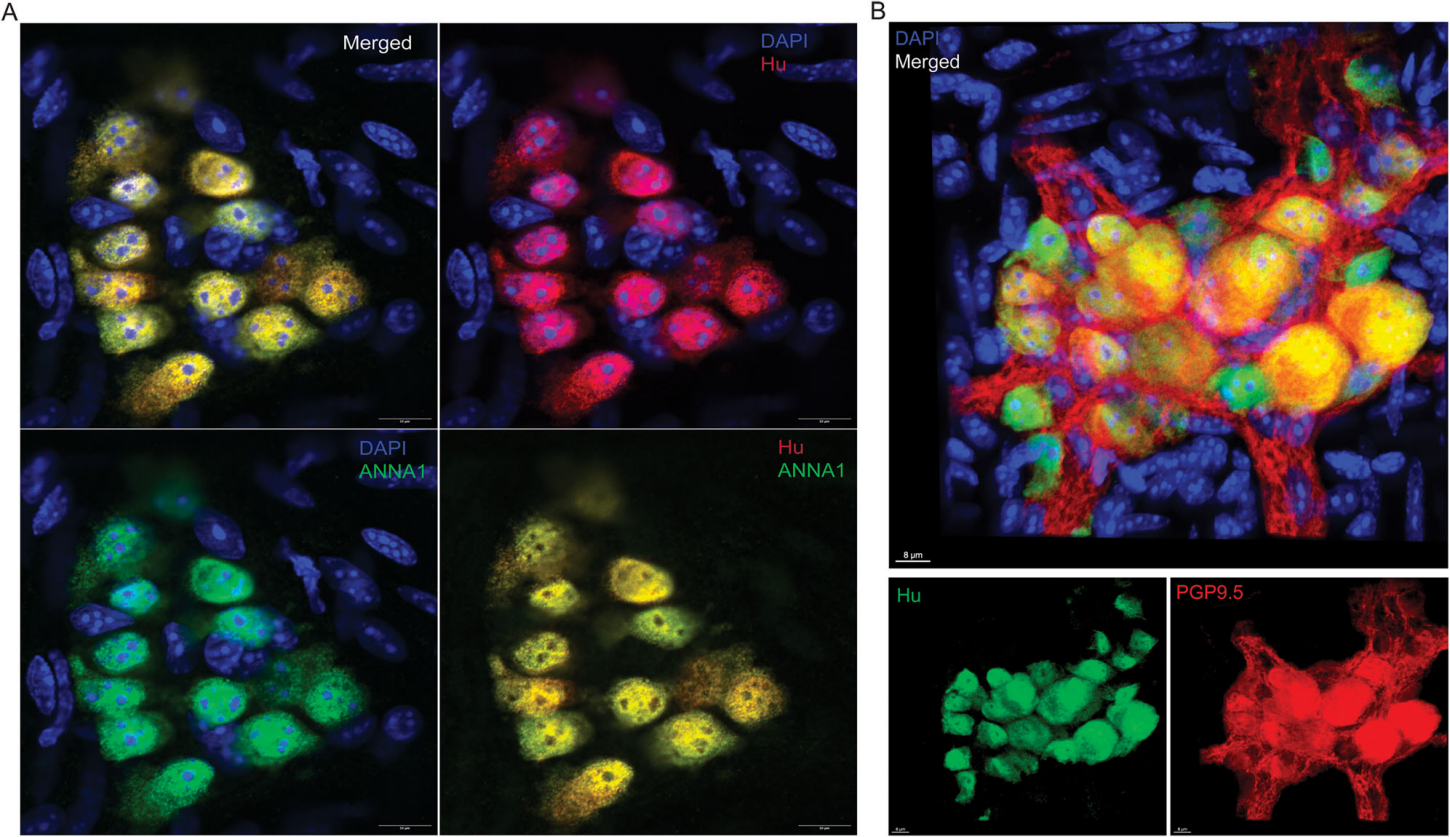
Hu-expression labels all myenteric ganglionic cells that express the pan-neuronal marker PGP9.5. ***A***, Representative image (merged and color-segregated) showing coimmunolabeling of an adult murine small intestinal myenteric ganglion with commercially available anti-Hu antibody (red) along with anti-Hu antibodies in the patient-derived ANNA1 antisera (green). Nuclei are labeled with DAPI (blue). Scale bar, 10 μm. ***B***, Representative image (merged and panel-segregated) showing coimmunolabeling of an adult murine small intestinal myenteric ganglion with commercially available antibodies against Hu (green) and PGP9.5 (red). Nuclei are labeled with DAPI (blue). Scale bar, 8 μm.

**Figure 2.**
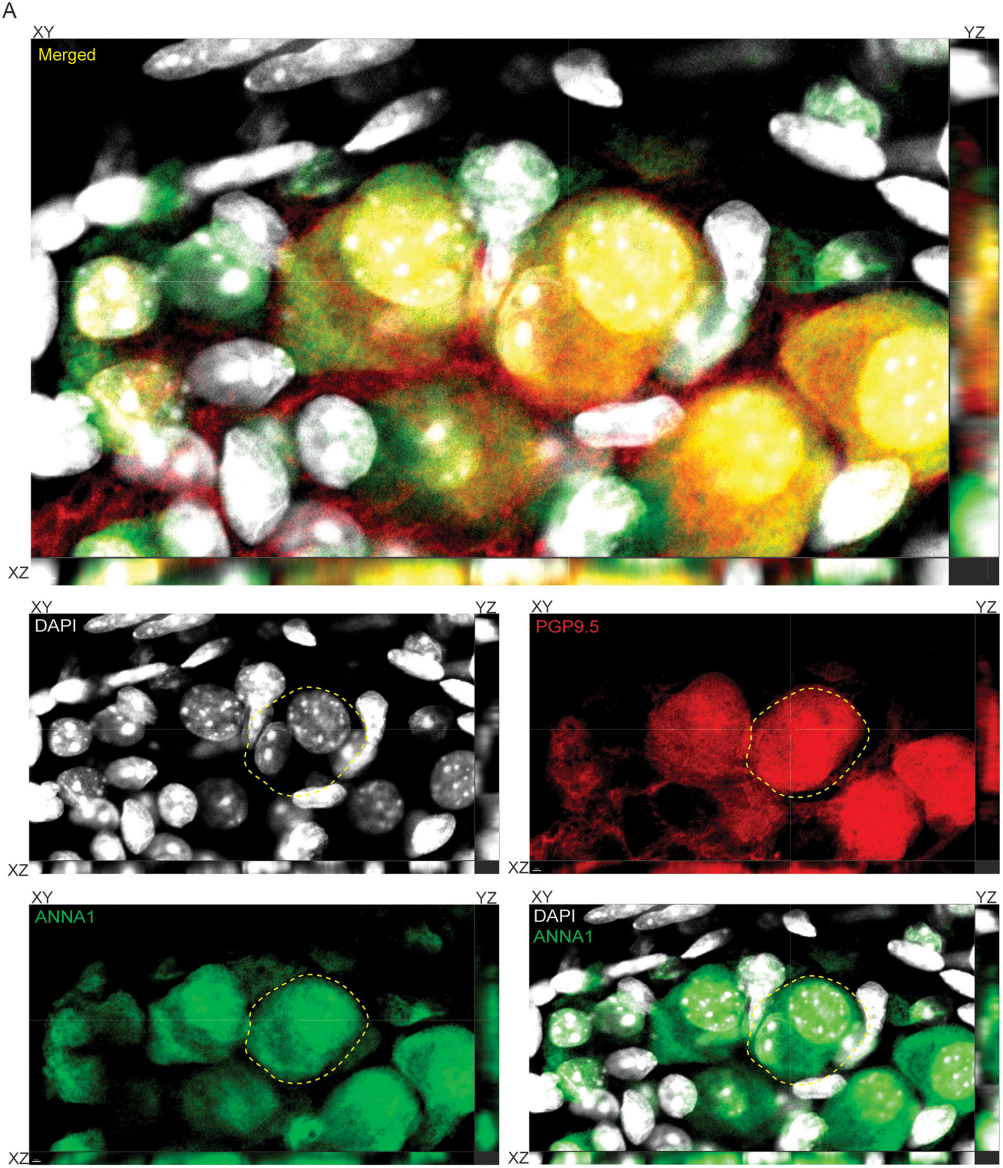
Binucleated ganglionic cells express Hu and PGP9.5. Orthogonal (merged and color-segregated) views of a 3D confocal microscopy image showing that a DAPI-stained- (gray), Hu- (green), and PGP9.5- (red) immunolabeled cell contains two nuclei. XY, YZ, and XY planes of the orthogonal views are denoted for every merged and panel-segregated image. Scale bar, 2 μm. Additional Hu-immunolabeled binucleated cell with conjoined nuclei is shown in Extended Data Figure 2-1.

Hu-expression is known to be a pan-neuronal marker for all adult enteric neurons (Liu et al., 2009; Desmet et al., 2014; Kulkarni et al., 2017, 2023; D'Errico et al., 2018; Kobayashi et al., 2021). These studies have used both commercially available antibodies against the neuronal Hu antigens (HuB/C/D), as well as the patient-derived ANNA1 sera which contain autoantibodies against the neuronal Hu antigens. As these are often used as alternatives, we tested whether they detected the same ganglionic cells and found by coimmunostaining that the commercially available anti-Hu antibodies detect the same cells as the anti-Hu autoantibodies in the patient-derived ANNA1 antisera (Fig. 1*A*). Next, we tested the coexpression of Hu and the pan-neuronal marker PGP9.5 and found that Hu immunostaining (ANNA1) colocalized with PGP9.5 immunostaining in the adult murine small intestinal MP (Fig. 1*B*). By imaging three-dimensional (3D) *z*-stack of a Hu and PGP9.5-immunostained adult murine small intestinal myenteric ganglia, we found that that some Hu^+^ PGP9.5^+^ cells within myenteric ganglia contained more than one nucleus (Fig. 2). This observation was reproduced in tissues from another mouse where two nuclei were observed in a single Hu-expressing cell (Extended Data Fig. 2-1).

10.1523/ENEURO.0005-24.2025.f2-1 Figure 2-1 **Observation of bi-nucleated or conjoined nuclei in Hu^+^ myenteric cells.** Orthogonal color-merged and color-segregated views of an image of a myenteric ganglion from an adult murine small intestinal tissue, where the tissue is immunolabeled with antibodies against Hu (green) and stained with nuclear dye DAPI (grey) shows the presence of two near or conjoined nuclei # and + in a contiguous Hu-immunolabeled cell. Scale bar denotes 1  µm. Download Figure 2-1, TIF file.

### About 10% of all adult murine small intestinal myenteric Hu-expressing cells show evidence of more than 2N DNA content by flow cytometry

**Figure 3.**
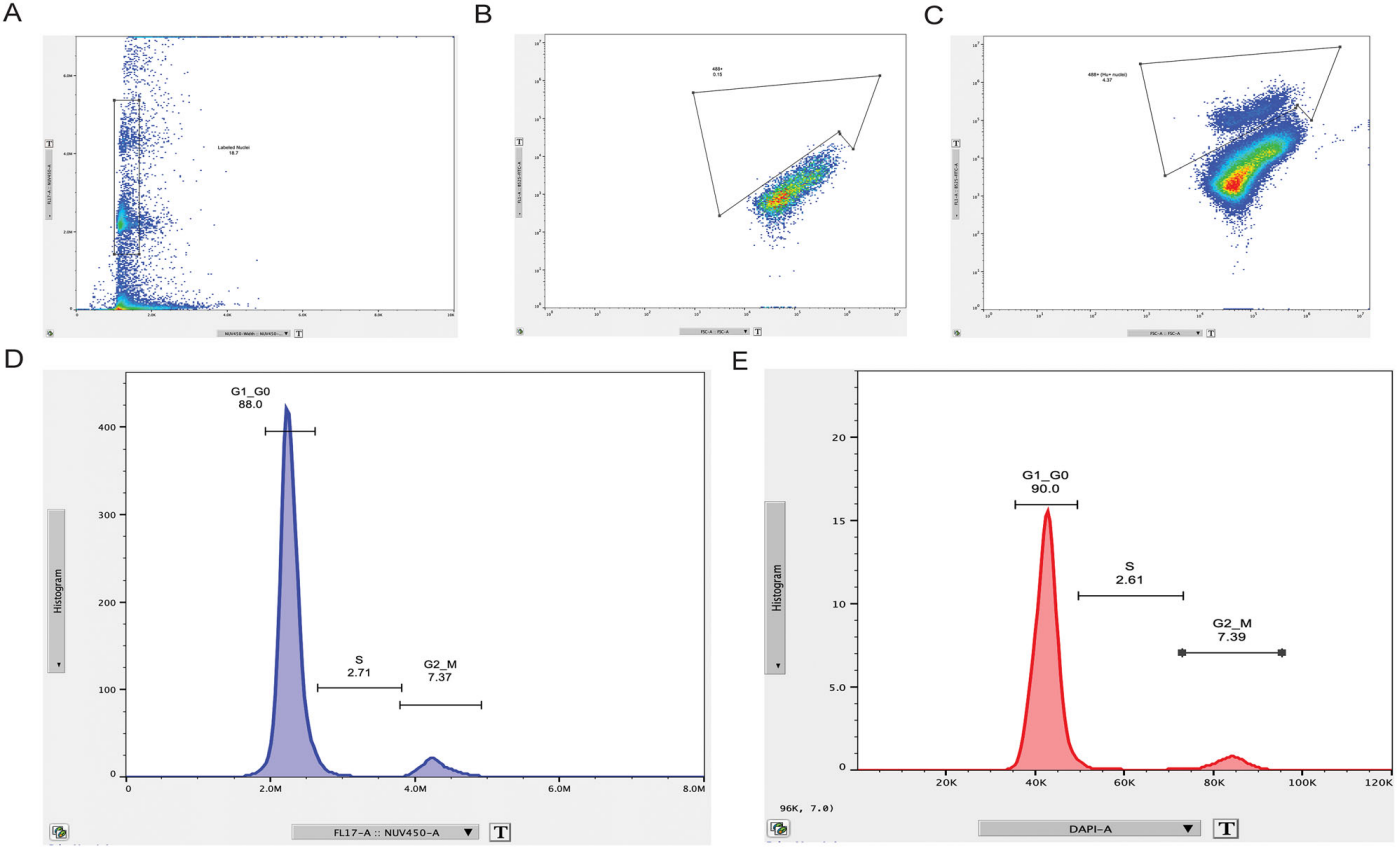
Evidence of S and G2/M phases of cell cycle in a population of Hu-immunolabeled cells from the small intestinal MP tissue of adult healthy mice. Flow analyses of nuclei isolated from the LM–MP tissue from adult healthy mice housed in a barrier facility at BIDMC when immunolabeled with directly conjugated anti-Hu 488 antibody and costained with a DNA-binding dye NucBlue show that (***A***) significant proportion of events present with detectable NucBlue-labeling and hence are annotated as nuclei. ***B***, Using a Hu-unstained population of NucBlue-labeled nuclei, we create gates for Hu-immunolabeled cells, which (***C***) contain Hu-labeled nuclei in a sample that was immunolabeled with a directly conjugated Hu antibody. ***D***, This population of isolated Hu- and NucBlue-labeled nuclei from adult murine small intestinal LM–MP tissues from mice from the BIDMC mouse colony, when analyzed using fluorescence intensity of NucBlue as a marker for DNA content, showed that while 88.0% of nuclei have DNA content (2N) expected in the G1 phase cells, 2.71% of Hu^+^ nuclei contain DNA content that corresponds to cells in S-phase (DNA replication phase), and 7.37% of Hu^+^ nuclei contain DNA content that corresponds to cells in G2/M phase (mitotic phase). The enumeration of nuclei does not include those that show less than 2N DNA content. ***E***, Flow analysis of nuclei isolated from LM–MP of similarly aged mice housed at JHU, which were immunolabeled with anti-Hu 647 antibody postfixation and were stained with the DNA dye DAPI, shows the presence of 90.0% of nuclei have DNA content (2N) expected in the G1 phase cells, 2.61% of Hu^+^ nuclei contain DNA content that corresponds to cells in S-phase (DNA replication phase), and 7.39% of Hu^+^ nuclei contain DNA content that corresponds to cells in G2/M phase (mitotic phase). Flow analyses gates for DAPI-labeled and Hu-immunolabeled nuclei from experiment performed at JHU are shown in Extended Data Figure 3-1.

While binucleated cells suggest impending cytokinesis in a cell (Panet et al., 2015), to further study whether cell cycling occurs in Hu-expressing cells, we first used flow cytometry-based estimation of DNA content in adult murine small intestinal Hu-immunolabeled nuclei to test whether myenteric Hu^+^ nuclei show presence of higher than the expected 2N DNA content, which would suggest the presence of cells in S-phase (DNA replication) and in G2/M phase (mitosis) of the cell cycle. On performing this assay on >10,000 unfixed Hu-immunolabeled nuclei derived from LM–MP tissues from adult healthy mice that were housed in the barrier facility at BIDMC, we observed that ∼10% of these Hu^+^ nuclei show evidence of DNA content greater than expected euploidy (Fig. 3*A–C*). Based on DNA content, 2.71% of all Hu^+^ nuclei were found to be in S-phase, and 7.37% were found to be in G2/M phase of the cell cycle. We next tested whether the observation of >2N DNA content in myenteric Hu^+^ nuclei was dependent on fixation or housing conditions. For this, Hu-immunolabeled fixed nuclei that were derived from the small intestinal LM–MP tissue of adult healthy mice housed in a nonbarrier facility at JHU were analyzed and were again found to have 2.61% of all Hu^+^ nuclei that were found to be in S-phase, and 7.39% were found to be in G2/M phase of the cell cycle (Fig. 3*D*; Extended Data Fig. 3-1). Thus, we establish a high degree of concordance in the proportions of Hu^+^ cells in S and G2/M phase of cell cycle in adult mice, irrespective of their housing conditions and colony location, as well as whether the nuclei were stained with or without fixation.

10.1523/ENEURO.0005-24.2025.f3-1 Figure 3-1 **Flow gates used for assessing Hu-immunolabeled nuclei from adult small intestinal LM-MP tissues from mice in the Johns Hopkins colony**. Fixed nuclei isolated from adult murine small intestinal LM-MP were stained with nuclear dye DAPI and directly conjugated Hu antibody (Alexa 647) and assessed to establish the gates for DAPI + nuclei (left plot), and DAPI + nuclei that immunolabeled for Hu (right plot). Download Figure 3-1, TIF file.

### About 10% of all adult murine small intestinal myenteric Hu-expressing cells express the cell cycle marker pH3

**Figure 4.**
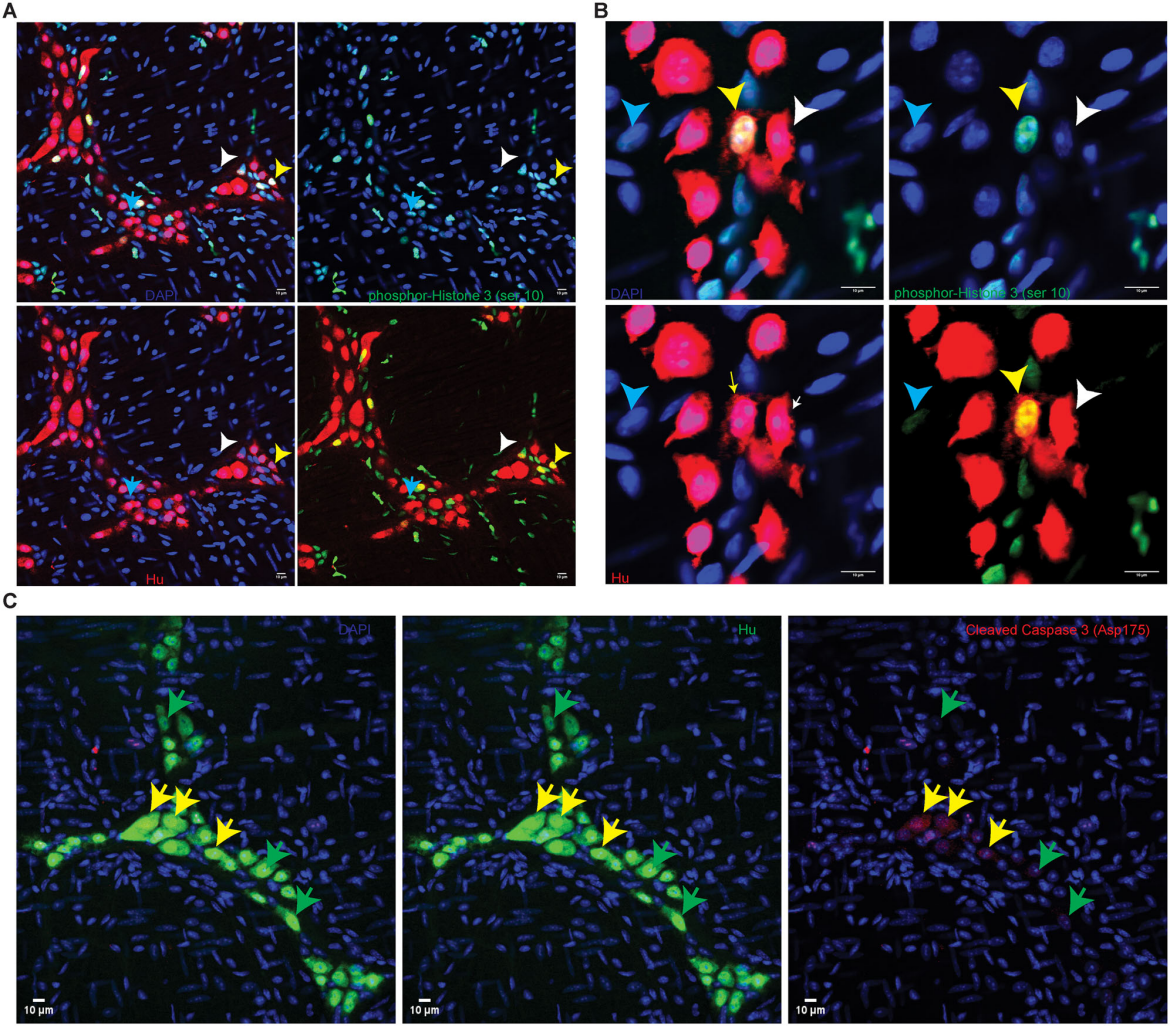
Cell proliferation marker pH3 is detected in neurons and other cells of the adult healthy MP tissue. Confocal microscopy shows that the (***A***) LM–MP tissue from an adult healthy mouse that was housed at JHU, when stained with unconjugated antibodies against pH3 (red) and the pan-neuronal marker Hu (green), shows the presence of pH3 immunoreactivity in ganglionic cells that do express Hu (yellow arrow) and those that do not express Hu (cyan arrow). pH3 immunoreactivity is also detected in extraganglionic cells in this tissue (white arrow) that are presumed to be LM cells. ***B***, Magnified image of a myenteric ganglia labeled with antibodies against pH3 (red) and Hu (green) again shows the presence of pH3-immunoreactive Hu–immunolabeled newborn neurons (yellow arrow), along with neurons that are not immunoreactive against pH3 (white arrow) and pH3-immunoreactive extraganglionic cells (cyan arrow). ***C***, Left, Representative image of the LM–MP tissue from an adult mouse housed in the barrier facility at BIDMC when stained with directly conjugated antibody against pH3 (red) shows the presence of significant pH3 immunoreactivity in the tissue. The tissue was also coimmunostained with directly conjugated anti-Hu antibody and the region of interest (white box) when magnified shows (right) the presence of Hu-immunolabeled (green) neurons that colabel for pH3. Nuclei are stained with DAPI (blue). ***D***, The representative image of the adult murine small intestinal LM–MP tissue, when stained with antibodies against CC3 (red) and the pan-neuronal marker Hu (green), shows the presence of CC3 immunoreactivity in a subset of Hu-immunoreactive neurons (yellow arrows), while other neurons in the same and other ganglia do not immunolabel for CC3 (green arrow). Nuclei are labeled with DAPI (blue). Scale bar, 10 µm.

**Figure 5.**
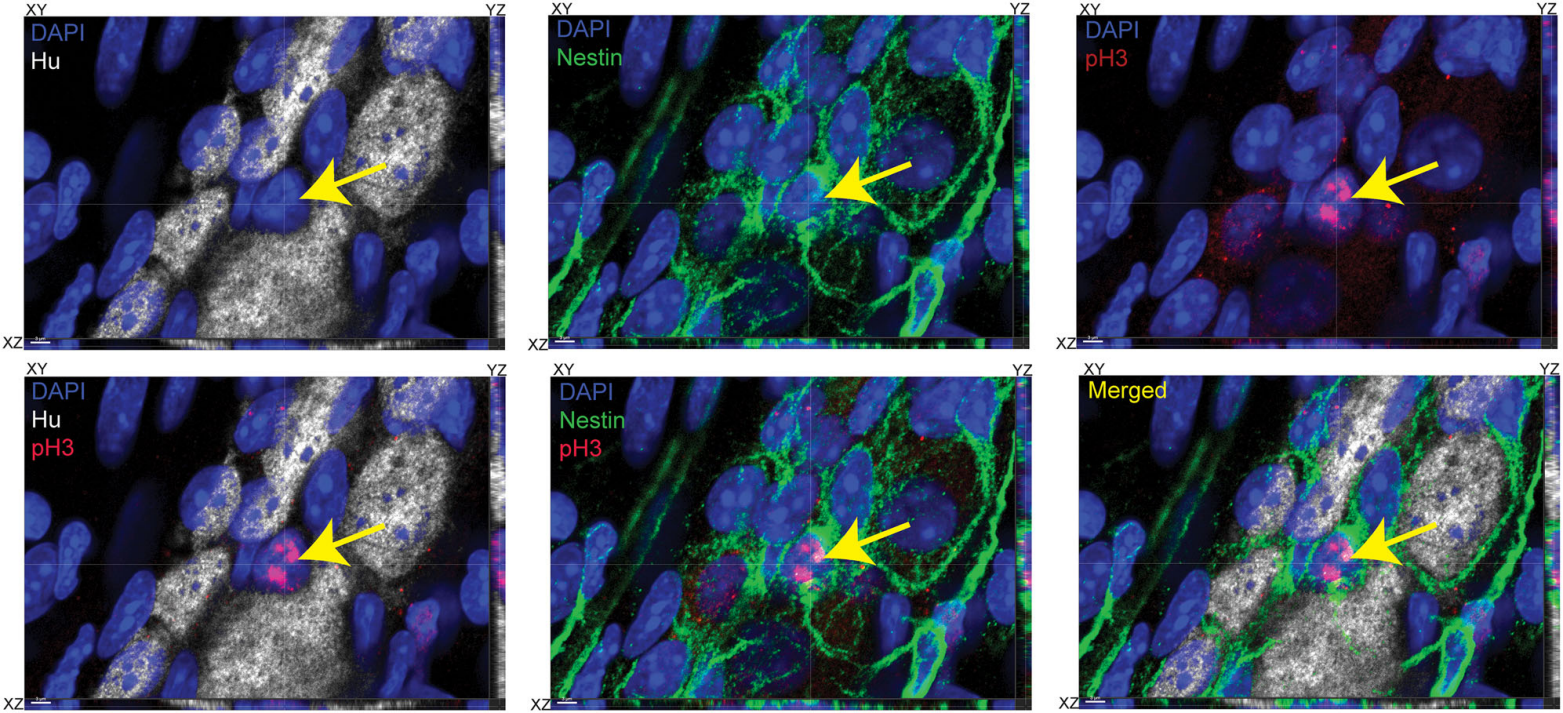
Nestin-expressing and Hu-nonexpressing ENPC express cell cycle marker pH3. Orthogonal (merged and color-segregated) views of a 3D confocal microscopy image showing a myenteric ganglion from an adult murine small intestinal tissue, where the tissue is immunolabeled with antibodies against Hu (gray), pH3 (red), and Nestin (green) and is stained with nuclear dye DAPI (blue). Yellow arrow points out a Nestin- and pH3-immunolabeled cell that does not immunolabel for Hu. The presence of pH3 immunoreactivity and the absence of Hu immunoreactivity in this Nestin-immunolabeled cell suggest it is a cycling enteric neural precursor cell. XY, YZ, and XY planes of the orthogonal views are denoted for every merged and panel-segregated image. Scale bar, 3 μm.

**Figure 6.**
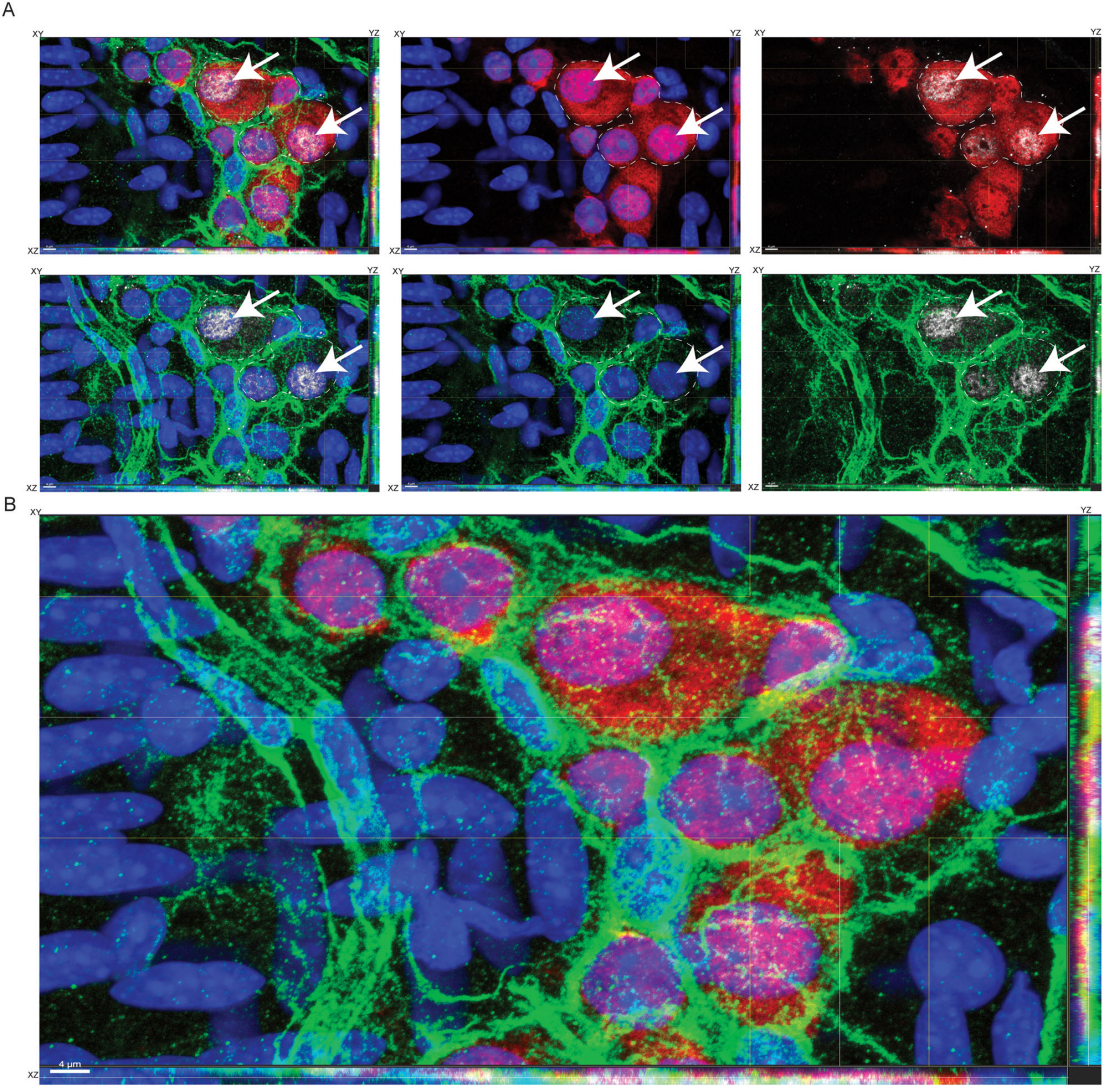
Hu-expressing cells that express cell cycle marker pH3 also exhibit Nestin immunoreactivity. Orthogonal (***A***) color-segregated and (***B***) color-merged image of a myenteric ganglion from an adult murine small intestinal tissue, where the tissue is immunolabeled with antibodies against Hu (red), pH3 (gray), and Nestin (green) and is stained with nuclear dye DAPI (blue). Dashed white lines depict a multinucleated contiguous Hu-immunolabeled cell, with white arrows showing that two of the three nuclei are immunolabeled with antibodies against pH3. The pH3^+^ Hu^+^ cells (white arrows) also show positive immunostaining for Nestin. XY, YZ, and XY planes of the orthogonal views are denoted for every merged and color-segregated image. Scale bar, 4 μm.

In addition to flow analyses of Hu-immunolabeled nuclei, we interrogated whether cycling Hu^+^ cells can be detected in tissues using a known cell cycling marker. For this, we further tested the presence of mitosis in adult enteric Hu^+^ cells by microscopic detection of an established mitotic marker pH3 in the neurons of the MP (Kim et al., 2007; Uguen et al., 2015; Tiede et al., 2018; Jiang et al., 2019; Rotelli et al., 2019). By using antibodies against the protein pH3 (phosphorylated at residue Ser10, which occurs at the initiation of the G2 phase and continues into metaphase), we observed that the adult healthy small intestinal LM–MP tissue derived from mice housed in the JHU mouse colony contains diverse cell populations of pH3-immunostained cells that are present both within and outside of the myenteric ganglia (Fig. 4*A*,*B*). Importantly, using the unconjugated pH3 antibody and the anti-Hu ANNA-1 antisera, we observe that the pH3-positive, intraganglionic cell population comprises of Hu-immunostained cells (Fig. 4*A*,*B*), as well as other unlabeled cells that we assume to be populations of proliferating enteric glial cells (Zeisel et al., 2018) and enteric neuronal precursor cells, that we have previously shown to express Nestin and Ki-67 (Kulkarni et al., 2017). Using conjugated pH3 and Hu antibodies, we tested whether adult myenteric ganglia in mice housed in the barrier facility at BIDMC also similarly contained pH3-immunoreactive Hu^+^ cells (Fig. 4*C*). Similar to earlier observations (Kulkarni et al., 2017), CC3 immunoreactivity, which indicates apoptotic cells, was found in a subset of myenteric Hu^+^ cells (Fig. 4*D*). We further tested whether pH3 immunoreactivity was found both in adult murine small intestinal enteric neural precursor cells (ENPC) that express Nestin but do not express Hu and in ganglionic cells that coexpress Nestin and Hu. By coimmunolabeling, we found that pH3 immunolabeling was found both in Nestin-expressing ENPC and in Hu-expressing cells that show detectable but low expression of Nestin (Figs. 5, 6). This suggests that Hu^+^ pH3^+^ ganglionic cells are distinct from Nestin-expressing cycling ENPC.

**Figure 7.**
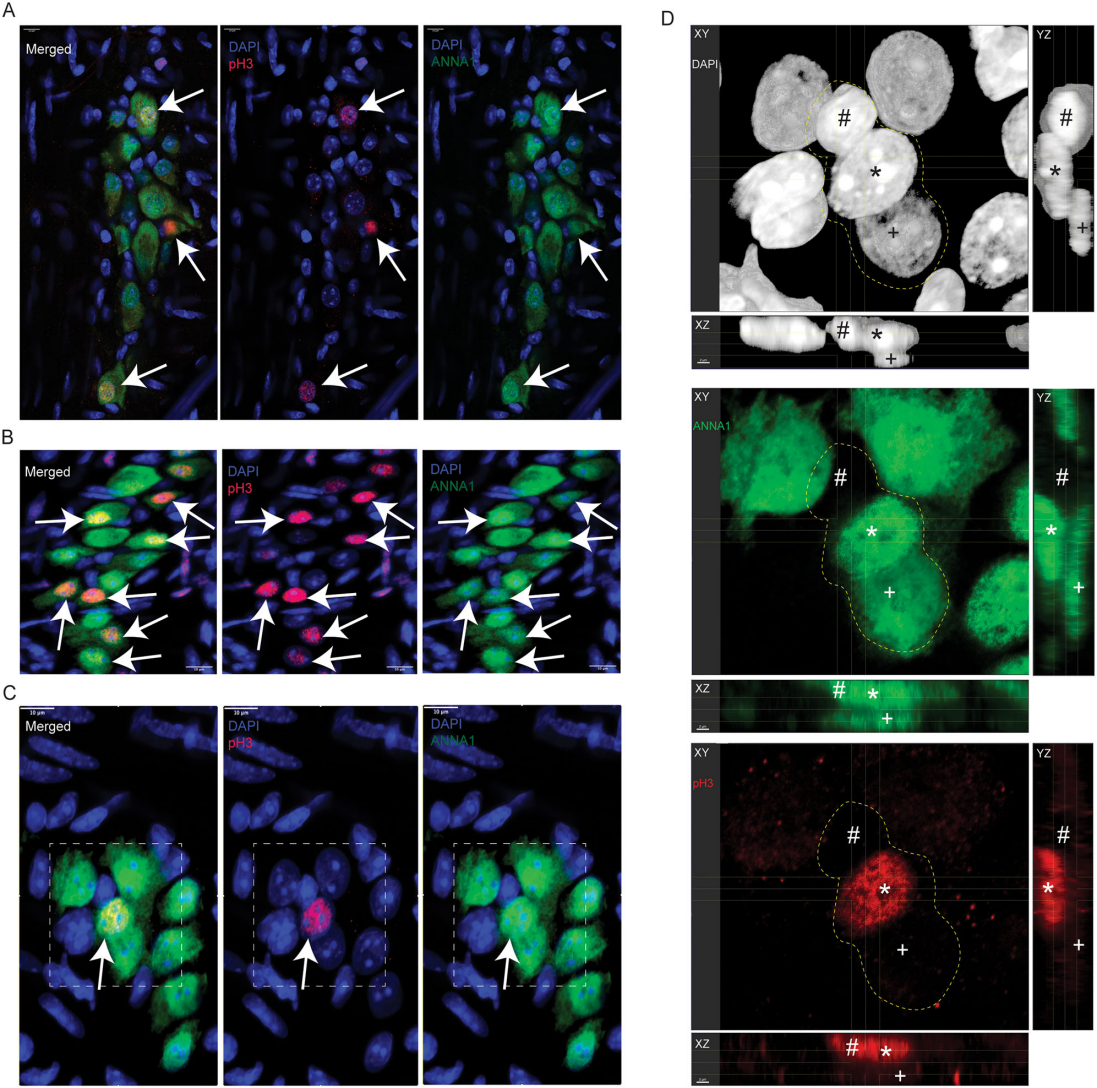
pH3 immunoreactivity in Hu^+^ cells is unaffected by fixation conditions. Immunostaining adult murine small intestinal LM–MP tissues that were fixed with 4% PFA for (***A***) 10 min, (***B***) 15 min, and (***C***) 20 min and subsequently immunostained with anti-Hu antibodies in ANNA1 serum (green) and pH3 (red) and counterstained with DAPI (blue). Merged and color-segregated views are shown for each of the treatments. White arrows show the presence of pH3 immunoreactivity in the DAPI-stained nuclei of Hu-immunoreactive cells in each of the treatments. White square in panel ***C*** is magnified in ***D*** and viewed in orthogonal views where XY, YZ, and XZ planes are shown. A contiguous nuclear structure (stained with DAPI, gray) containing three nuclear lobes “#, *, +” are observed within dashed white lines. The nuclear lobe “#” is unstained by pH3 (red) and ANNA1 (green) antibodies, lobe “*” is stained by both pH3 and ANNA1, and lobe “+” is stained by ANNA1 but not by pH3. The three panels show color-segregated views depicting an asymmetric cellular structure where parts of the nuclear structure exhibit Hu and/or pH3 immunoreactivity, while another part does not. XY, YZ, and XY planes of the orthogonal views are denoted for every merged and color-segregated image. Scale bars: ***A–C***, 10 μm; ***D***, 2 μm.

We previously reasoned that isolated adult murine small intestinal LM–MP tissues require gentle fixation (Kulkarni and Pasricha, 2022; Kulkarni et al., 2023). To test whether the presence of pH3 in Hu^+^ ganglionic cells is an artifact of gentle fixation, we tested three additional timepoints of 10, 15, and 20 min of fixation and found that pH3 immunoreactivity was present in Hu-immunolabeled cells at each of these timepoints. Similar to our observations on binucleated Hu^+^ cells in gently fixed tissues, tissues fixed for 20 min again showed the presence of binucleated Hu^+^ cells that showed evidence of asymmetric cell division (Fig. 7).

**Figure 8.**
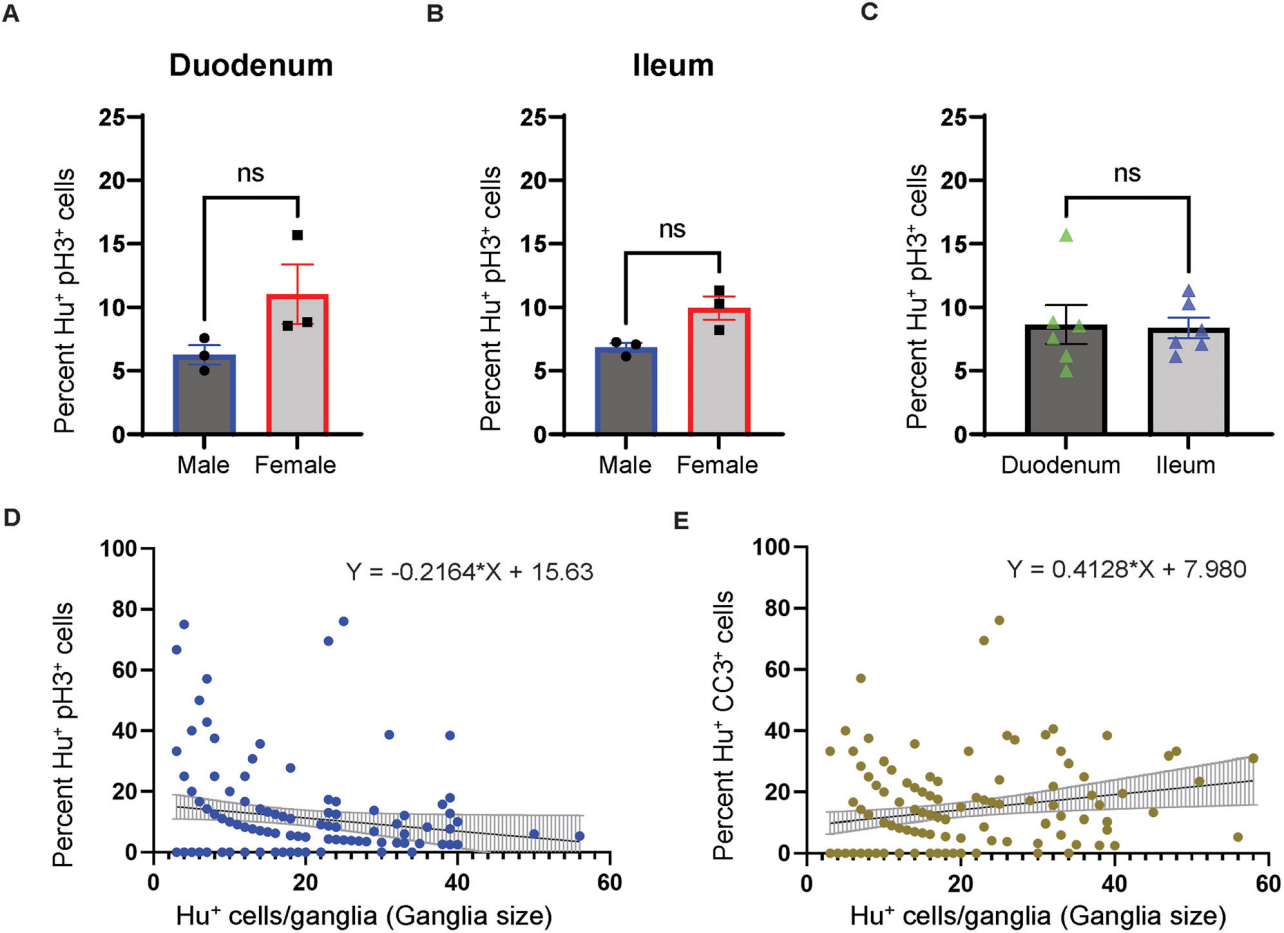
Quantifications of pH3-immunoreactive and CC3-immunoreactive Hu^+^ cells in the adult murine gut. ***A***, Quantification of pH3-immunoreactive Hu^+^ cells in the duodenal tissue of age-matched male and female mice show no significant sex bias in their proportions (*p* > 0.05, Students’ *t* test). ***B***, Quantification of pH3-immunoreactive Hu^+^ cells in the ileal tissue of age-matched male and female mice show no significant sex bias in their proportions (*p* > 0.05, Students’ *t* test). ***C***, Quantification of pH3-immunoreactive Hu^+^ cells in the duodenal and ileal tissue of age-matched mice show no significant differences in their proportions between the two tissue regions (*p* > 0.05, Students’ *t* test). ***D***, Distribution of the percentage of pH3-immunoreactive Hu^+^ cells in small intestinal ganglia containing various numbers of Hu^+^ cells. ***E***, Cubic curve fit (black line) for the distribution of CC3-immunoreactive Hu^+^ cells in small intestinal ganglia containing various numbers of Hu^+^ cells.

Since coimmunolabeling with antibodies against pH3 and pan-neuronal marker Hu provides evidence of mitosis in adult small intestinal myenteric Hu^+^ cells, we next quantified the proportions of pH3-immunoreactive mitotic small intestinal myenteric Hu^+^ cells at steady state in adult mice. By confocal microscopy, we observed 2,577 Hu-immunolabeled Hu^+^ cells from the small intestine of six adult healthy male and female mice (*n* = 3 of either sex), which included the MP tissue from both duodenum and ileum. We found that 8.54% ± 1.09 (mean ± SEM) of all Hu-immunolabeled cells also showed coimmunolabeling with anti-pH3 antibodies (Fig. 8*A*,*B*).

Next, we analyzed whether pH3 immunoreactivity in Hu^+^ cells from ileal or duodenal myenteric ganglia showed any sex bias. In the duodenal myenteric ganglia, we counted 670 Hu^+^ cells in tissues from female mice and 601 Hu^+^ cells in the tissues from male mice (*n* = 3 of either sex) and found no significant difference between the percentage of pH3-immunoreactive Hu^+^ cells between the two sexes (mean ± SEM pH3^+^ neurons, female mice, 11.63 ± 2.00; male mice, 6.26 ± 0.75; *p* = 0.17; unpaired *t* test with Welch's correction; Fig. 8*A*). In myenteric ganglia of the ileum, we counted 667 Hu^+^ cells in the tissues from female mice and 639 Hu^+^ cells in the tissues from male mice (*n* = 3 of either sex) and found no significant difference between the percentage of pH3-immunoreactive Hu^+^ cells between the two sexes (mean ± SEM pH3^+^ neurons, female mice, 9.93 ± 0.92; male mice, 6.82 ± 0.34; *p* = 0.06; unpaired *t* test with Welch's correction; Fig. 8*B*). Finally, we compared pH3 immunoreactivity in populations of Hu^+^ cells between ileal and duodenal tissue across the two sexes and found no significant difference (duodenal tissue, Hu^+^ cells counted, 1,271; mean ± SEM pH3^+^ Hu^+^ cells, 8.64 ± 1.52; ileal tissue, Hu^+^ cells counted, 1,306; mean ± SEM pH3^+^ Hu^+^ cells, 8.38 ± 0.82; *p* = 0.88; unpaired *t* test with Welch's correction; Fig. 8*C*).

We have previously shown that there is significant heterogeneity in ganglionic size, demonstrated by small intestinal myenteric ganglia containing as few as 3 neurons or over 100 neurons (Kobayashi et al., 2021). Here, we tested whether the proportion of mitotic Hu^+^ cells correlate with ganglionic size in the adult healthy small intestinal MP. By analyzing the proportions of pH3-immunoreactive Hu^+^ cells in 147 ganglia of various sizes (3–56 neurons per ganglia) from duodenum and ileum from six mice together, we found an absence of any significant correlation between the percentage of Hu^+^ cells immunoreactive for pH3 in myenteric ganglia and the numbers of Hu^+^ cells within ganglia (ganglionic size; D’Agonistino and Pearson’s test for normality showed non-normal distribution, nonparametric Spearman correlation test, *r* = −0.05625; *p* = 0.5; Fig. 8*D*).

We next tested whether there was any correlation between ganglionic size and the proportions of CC3-immunoreactive apoptotic Hu^+^ cells in the adult healthy murine small intestinal MP. We quantified the percentage of CC3-immunoreactive Hu^+^ cells from 83 myenteric ganglia from three mice, where the myenteric ganglionic size ranged from 3 to 58 neurons. We found that the proportion of CC3^+^ Hu^+^ cells in small intestinal myenteric ganglia increased significantly with ganglionic size (D’Agonistino and Pearson’s test for normality showed non-normal distribution, nonparametric Spearman correlation test, *r* = 0.4181; *p* < 0.0001; Fig. 8*E*).

### Human small intestinal myenteric ganglia show evidence of neuronal turnover

**Figure 9.**
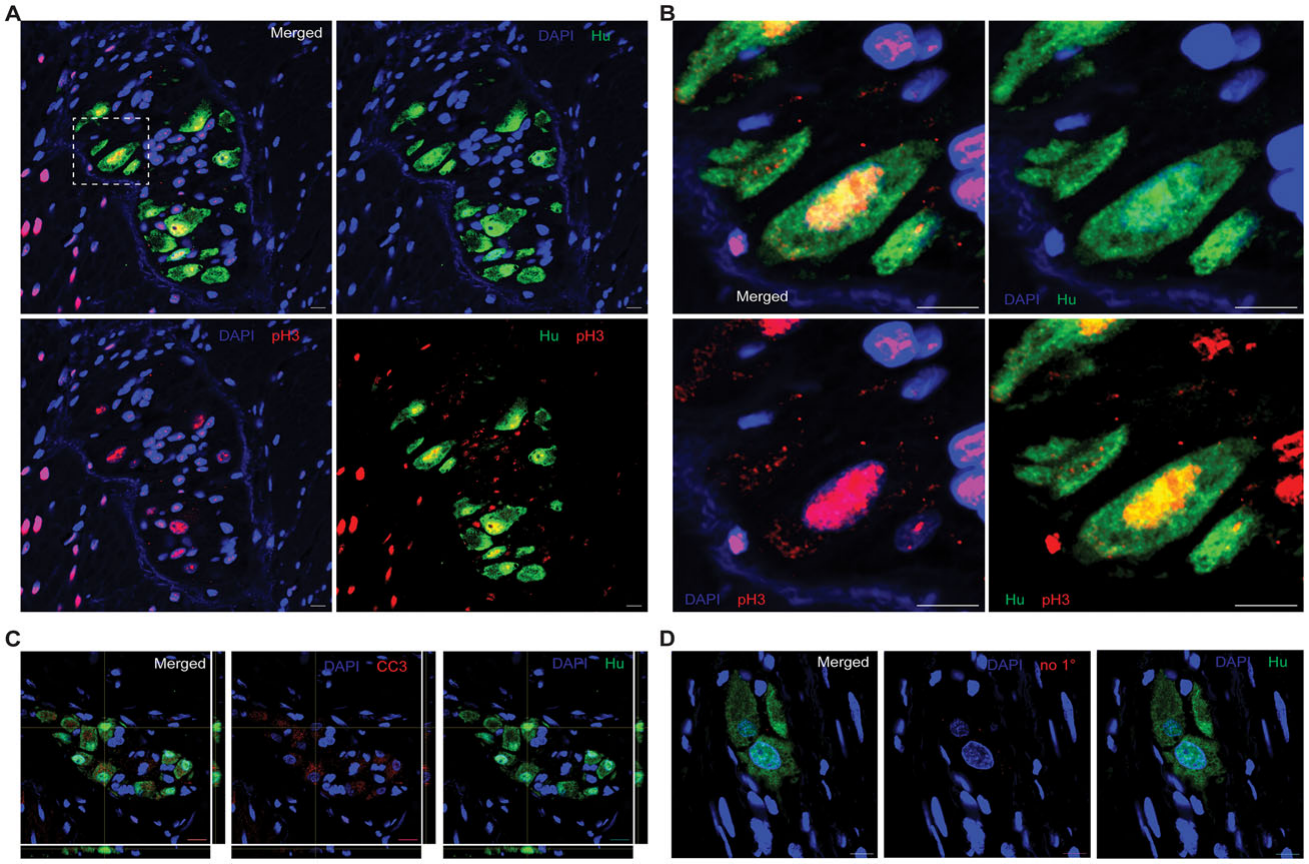
Hu^+^ myenteric cells expressing cell cycle marker pH3 or expressing apoptotic marker CC3 exist in human small intestinal myenteric ganglia. ***A***, Cells of the LM layer of the human gut wall in FFPE tissue sections from the adult human small intestine with immunolabeling of mitotic marker pH3 (red) and costaining with DAPI (blue) show detectable pH3 immunoreactivity in many cells of this tissue. Image created by stitching contiguous 40× images of the section which was imaged with the EVOS M7000 microscope. ***B***, Coimmunolabeling adjacent sections of this tissue with directly conjugated antibodies against mitotic marker pH3 (red) and pan-neuronal marker Hu (green) and by imaging them with the M7000 microscope, we observe the presence of pH3-immunoreactive cells that also immunolabel with antibodies against Hu (yellow arrows). ***C***, Confocal microscopy of FFPE human tissues that were immunolabeled with anti-Hu ANNA-1 antisera and anti-pH3 antibody and then suitably costained with secondary antibodies again shows the presence of pH3 and Hu colabeled cells within the myenteric ganglia (yellow arrow). ***D***, Orthogonal views generated from confocal microscopy of a myenteric ganglia immunostained with antibodies against CC3 (red) and anti-Hu ANNA-1 (green) show the presence of CC3-immunolabeled neurons. ***E***, Representative image of a myenteric ganglia immunostained with antibodies against Hu (green) but no primary antibodies for the red channel shows a lack of nonspecific staining with the secondary antibodies. Photomicrographs in panels ***C–E*** were generated by confocal microscopy. Nuclei are labeled with DAPI (blue); scale bar, 10 µm (***C–E***).

We next tested whether the adult human small intestinal MP tissue also shows evidence of significant cell proliferation through the presence of pH3-immunoreactive Hu^+^ cells. In immunolabeled FFPE tissue sections, by using directly conjugated antibody against pH3, we found that many cells in the LM layer of human gut wall also showed immunoreactivity against the mitosis marker pH3 (Fig. 9*A*). By coimmunostaining these FFPE tissue sections with antibodies against pH3 and Hu, we found that many pH3-immunoreactive cells were found in the myenteric ganglia, some of which were also immmunolabeled by antibodies against Hu (Fig. 9*B*). We enumerated proportions of pH3^+^ Hu^+^ cells in myenteric ganglia in FFPE tissue sections from duodenal full-thickness tissues derived from four human samples. We counted a total of 152 Hu^+^ ganglionic cells [mean ± standard error (SE) of Hu^+^ cells counted per sample, 37.5 ± 9.56] and found that 23.43 ± 5.22% (mean ± SE) of Hu^+^ cells is also immunolabeled for the mitosis marker pH3. These data provide evidence that significant proportions of human Hu^+^ myenteric ganglionic cells are mitotic in nature.

Similarly, we also found that the apoptotic marker CC3 was detected in a subset of human small intestinal myenteric neurons (Fig. 9*C*), while the negative control for CC3 immunoreactivity (performed without primary antibody) showed no background signal (Fig. 9*D*).

## Discussion

Using established microscopic, flow cytometric, and immunolabeling-based assays (Kim et al., 2007; Kim and Sederstrom, 2015; Uguen et al., 2015; Alonso-Martin et al., 2018; An et al., 2018; Tiede et al., 2018; Jiang et al., 2019; Rotelli et al., 2019), our report provides evidence that a significant proportion of Hu^+^ ganglionic cells in the adult small intestinal MP tissue undergo mitosis. Neurogenesis from precursor cells requires DNA replication and mitosis, and the detection of mitosis and apoptosis in Hu-immunolabeled cells in healthy small intestinal MP tissue shows that there is ongoing genesis of neurons in this organ, which offsets the continual loss of neurons to apoptosis. Our prior study, which used thymidine analog-based assays to demonstrate that almost 90% of adult small intestinal neurons were generated during a 2 week period, had suggested that ∼10% of neurons are generated (and hence are newborn) on any given day (Kulkarni et al., 2017). Here, our DNA content-based flow cytometry analysis shows that ∼10% of Hu-immunolabeled myenteric nuclei showed evidence of DNA content associated with G2/M (or mitotic) phase of the cell cycle. This is consistent with our microscopy-based data that demonstrate the presence of binucleate Hu^+^ cells and the immunolabeling-based data that show that ∼10% of Hu-immunolabeled cells within myenteric ganglia express the mitotic marker pH3. Thus, our observation of ongoing mitosis in Hu-immunolabeled cells using these different methods closely matches the rate of neurogenesis we previously deduced using detection of thymidine analogs (Kulkarni et al., 2017). In addition, the presence of pH3 can be readily detected in >20% of Hu-immunolabeled ganglionic cells in the adult human gut—further suggesting that a similar neurogenic process is also present in the adult human gut. We again confirm that a subpopulation of adult murine small intestinal myenteric neurons showed detectable presence of the apoptotic marker CC3 (Kulkarni et al., 2017).

Evidence of DNA replication (S-phase) and mitosis (G2/M phase) in Hu-immunolabeled cells suggests that in addition to being a marker for mature functional neurons, Hu is also expressed by cycling neuroblasts or transit amplifying cells that are committed to a neuronal fate. Thus, rather than suggesting that adult enteric neurons are mitotic in nature, we infer that Hu immunoreactivity is not restricted to mature neurons. This argument is supported by prior studies that have shown that Hu is expressed in neuronally committed cycling progenitors or neuroblasts as well as in immature neurons (Ekonomou et al., 2015; Seki et al., 2019). The presence of a significant population of cycling Hu^+^ neuroblasts in the adult MP provides evidence of a robust steady-state neurogenic process in the adult gut.

The thymidine analog-based assay we previously deployed to observe adult enteric neurogenesis requires complicated methods (Kulkarni and Pasricha, 2022). In addition, by virtue of being based on the uptake, incorporation, and accumulation of these chemicals over time into newly synthesized nucleic acids, this assay can neither sensitively measure the ongoing rate of DNA synthesis and cell proliferation at any given point of time, nor can it be used to assess the rate of neurogenesis in humans. With flow analyses, immunolabeling, and microscopy-based methods, we use simple established assays to robustly measure the proportions of cycling neuroblasts in the tissue to infer the rate of neurogenesis at health in the adult human and murine gut.

Furthermore, two previous studies suggested that the proportions of apoptotic myenteric neurons are significantly greater in the human gut than in the murine gut (human gut 20–30% apoptotic neurons vs 10–11% in murine gut; Kozlowska et al., 2016, 2018; Kulkarni et al., 2017). Thus, it would be expected that the rate of genesis of neurons in the adult human gut would also be greater than in the murine gut. Our results, which show that ∼23–24% of all human myenteric Hu^+^ cells immunolabel for the mitotic marker pH3, closely match the published rate of apoptosis in human ENS neurons. These data demonstrate that as in the murine gut, the proportions of newborn and apoptotic neurons in the human gut are evenly matched. This suggests that as established by us previously in mice, continual neurogenesis and neuronal loss are also a feature of the adult human ENS.

The immunofluorescence analyses of mitotic neuroblasts and apoptotic neurons in the murine small intestinal MP also allowed us to test whether neurogenesis occurs equally in all myenteric ganglia across two different small intestinal regions and between the two sexes. Taking the proportions of mitotic neuroblasts to mature neurons as a measure of the neurogenic activity for each ganglion, we provide evidence that neurogenic activity does not significantly differ between differently sized ganglia, between two different intestinal regions, or between sexes. However, while the rate of neurogenesis does not change across ganglia, we observe that larger ganglia (i.e., with greater number of neurons) have a higher percentage of neurons undergoing apoptosis. If neurogenesis occurs at a uniform rate across all ganglia, then the observation that apoptosis occurs more frequently in larger ganglia implies that there may be a regulatory process that discourages the preservation or further enlargement of these larger ganglia. This is reflected in our prior findings that showed that smaller ganglia are much more frequent than larger ganglia in the adult small intestinal MP tissue (Kobayashi et al., 2021). This suggests that the balance between the genesis and death of neurons within ganglia is crucial for regulating ganglionic size. Furthermore, it would indicate that any disruptions to this balance, whether through increased neurogenesis or elevated neuronal death, could alter the normal proportions of smaller and larger ganglia, potentially leading to disorders in intestinal movement. While whether such a ganglionic size-associated skew in genesis and loss of neurons extends to human tissue is still unknown, it was recently demonstrated (Boschetti et al., 2019) that patients with dysmotility have significantly reduced neuronal numbers in myenteric ganglia (or that the presence of larger ganglia in their ENS is greatly reduced). These are similar to other observations (Bernardini et al., 2012) that observed fewer numbers of myenteric neurons in patients with ulcerative colitis than in healthy controls. These observations suggest that the ganglionic rate of neurogenesis and of neuronal loss may change in disease to alter the ENS structure.

That adult enteric neurogenesis at steady state occurs in the adult ENS has been controversial (Sharkey and Mawe, 2023). Despite our prior study that observed significant proportions of label-retaining myenteric neurons in mice dosed with thymidine analogs (Kulkarni et al., 2017), other investigators were unable to replicate these findings and detect thymidine analog-based label retention in neurons (Virtanen et al., 2022). While it may be argued that the inability to detect enteric neurogenesis at steady state in the adult healthy gut may be due to technical challenges and issues (Kulkarni and Pasricha, 2022), our study furthers the case for steady-state adult enteric neurogenesis by testing the presence of DNA synthesis and mitosis in Hu-expressing myenteric cells. A recent report studied adult enteric neurogenesis at health and in disease using a different immunohistochemical marker for newborn neurons Sox2, which was also found to label ∼10% of all Hu^+^ myenteric cells in the adult healthy colon (Miyata et al., 2024). The concordance between the proportions of Sox2^+^ Hu^+^ “newborn” neurons in the colon in their study and the proportions of pH3^+^ Hu^+^ myenteric cells and Hu^+^ cells in G2/M phase in our study provides evidence that myenteric neurons in the small intestine and colon do turn over in significant numbers, that the rate of neurogenesis or proportions of mitotic neuroblasts matches the observed rate of neuronal loss across various studies (Chandrasekharan et al., 2011; Nezami et al., 2014; De Schepper et al., 2018; Kulkarni et al., 2023), and that steady-state neurogenesis is an important mechanism through which adult ENS homeostasis is maintained (Kulkarni and Pasricha, 2022).

While this study furthers our model of continual neurogenesis to support neuronal homeostasis in the adult ENS, further work is needed to answer important questions that emerge considering this data and our other recent reports. One recent study provides evidence that the adult ENS consists of equal proportions of neurons derived from neural crest (NC) and mesodermal lineages (Kulkarni et al., 2023), and our prior study using thymidine analogs found that ∼90% of adult enteric neurons turn over during a 2 week period (Kulkarni et al., 2017). The near-comprehensive nature of neuronal turnover in the adult ENS suggests that in addition to genesis of NC-derived enteric neurons, which we showed occurs from Nestin-expressing NC–derived precursor cells, the population of mesoderm-derived enteric neurons (MENs) also turn over at steady state. While our report found molecular evidence of continual genesis of MENs in the postnatal ENS (Kulkarni et al., 2023), this current report does not test the identity of precursor cells for the MEN lineage or the proportions of mitotic neuroblasts of the two different lineages. Future work will focus on establishing how ganglionic Hu^+^ cells of the mesodermal and NC-lineage differ in their proportions of cycling cells at different ages. In addition, our microscopic assessment of myenteric ganglia provides evidence of binucleated cells and of pH3-immunolabeled Hu^+^ cells that appear to undergo asymmetric division into Hu^−/+^ cells. Mechanisms of cell division include canonical nucleokinesis and cytokinesis, as well as noncanonical endoreduplication and closed mitosis, through which cells may increase their nuclear DNA content without cytokinesis or maintain their nuclear membranes while undergoing cytokinesis (Shu et al., 2018; Rotelli et al., 2019; Dey et al., 2020). While the presence of asymmetric Hu-immunolabeled cells, where cell structure shows varying immunolabeling for Hu and pH3, suggests the presence of noncanonical cytokinetic mechanisms in the adult ENS, the nature of the precise cell biological mechanisms through which Hu^+^ cells cycle need to be further interrogated in detail. This study provides the rationale for studying these mechanisms in the future.

Our study is robust as it establishes concordance of our observations through different approaches—the high-resolution microscopy approach to test the presence of binucleated Hu-expressing cells, DNA content-based flow analyses to test the presence of diploid DNA content indicative of S- and G2/M phase, and the pH3-based immunofluorescence assay-based approach to identify and enumerate the proportions of mitotic Hu-expressing cells in adult murine and human ENS. Thus, this study further confirms the presence of the homeostatic mechanism of steady-state neuronal turnover in the adult ENS.

## Synthesis

Reviewing Editor: Deanna Smith, University of South Carolina Decisions are customarily a result of the Reviewing Editor and the peer reviewers coming together and discussing their recommendations until a consensus is reached. When revisions are invited, a fact-based synthesis statement explaining their decision and outlining what is needed to prepare a revision will be listed below. The following reviewer(s) agreed to reveal their identity: Keith Sharkey.

The authors responded comprehensively to the reviews and addressed the reviewers concerns.

## Author Response

The authors wish to thank the reviewers for their review, and in this revised manuscript, we have provided new data and analyses, revised prior analyses, and edited the manuscript suitably to respond constructively to the reviewers' comments. Succinctly, we have now included extensive confocal microscopy data that allow the interrogation of myenteric cells that are immunolabeled with antibodies against Hu, PGP9.5, phosphor-Histone H3 (pH3), and Nestin to show the presence binucleated cells that immunolabel for Hu and for PGP9.5, Hu+ cells with conjoined nuclei suggesting that they are in a state of cell division, presence of Nestin in Hu and pH3 immunolabeled cells, observations of asymmetric cell division. We also provide enumeration of pH3-immunoreactive Hu+ myenteric cells in the human gut and provide revised analyses of the flow cytometry data after extensive consultation with flow cytometry expert. Finally, we have edited the manuscript to ensure we do not overreach in our conclusions.

We request the reviewers to view the data presented in this manuscript in totality, as no single assay can provide us with an absolute unequivocal answer on the occurrence of adult enteric neurogenesis at steady state. Kulkarni et al, 2017 showed label retention in a set of Hu-expressing cells, and Kulkarni et al, 2023 used molecular analyses to provide evidence of cell cycling in neurons. However, to further interrogate the evidence supporting enteric neurogenesis in the healthy murine adult gut, we provide important datapoints here that together support the hypothesis that adult enteric neurogenesis is a mechanism to promote homeostasis in the adult ENS. Our argument in this manuscript depends on three main observations - the presence of pH3 (a validated marker for mitosis) in ~10% of Hu+ cells, the presence of >2N content of DNA in ~10% of Hu+ cells, and the observations of bi-nucleated cells that often show conjoined nuclei. Furthermore, our manuscript also shows that pH3-immunoreactive cells occur in the adult human gut. Recently, another publication in AJP-GI (which we cite) observes that ~10% of all adult colonic myenteric Hu+ cells express the newborn neuron/precursor marker Sox2 - which provides further support to our observations and hypothesis that adult ENS neurogenesis occurs at steady state at a significant rate.

In this response, we provide point-by-point response to your earlier thoughtful comments, and we sincerely hope that you would find our responses and our revised document worthy of acceptance and publication in the journal.

Reviewer 1 comments:

Adult enteric neurogenesis remains a very controversial topic, with one group demonstrating it (the current authors), but others (e.g., PMID: 35421596) denying its existence. The current article does not provide a balanced discussion of this topic by not citing evidence for and against it. The contentious nature of this debate in part provides a better rationale for their current studies, which appear to support their contention that there is a baseline level of adult neurogenesis. I recommend revising both the introduction and discussion to ensure there is a balanced account of this topic.

We thank the reviewer for their comment, and have suitably modified the Discussion section to include a paragraph on the controversial nature of adult enteric neurogenesis at health, and have discussed it in the context of our current data, prior data from other investigators, and a recent study from the Japanese group who show the presence of Sox2-expression (a marker for newborn neurons and neurogenic cells) in ~10% of all Hu-expressing cells in the healthy adult colonic LM-MP. We regret adding more to the introduction as we are bound by a strict word limit for this section.

It is incumbent upon authors who propose new approaches that demonstrate something controversial to validate their approaches where it is practical to do so. The authors show evidence for mitotic neuroblasts in the enteric nervous system, but do not demonstrate these techniques applied under the same conditions reveal expected results in the intestinal mucosa, where all authors agree on the presence and distribution of cell division. Such evidence would greatly strengthen this paper which otherwise lacks any sort of positive control for the labelling approaches that are used.

We thank the reviewer for their comment, but we humbly disagree that detection of phosphor-Histone H3 or the flow analyses to test for DNA content constitute novel approaches for detecting proliferating or mitotic cells. Both approaches were chosen precisely because they are well validated across a number of different cell types, organs, and species. In contrast to the earlier report by Kulkarni et al 2017, where the optimization of techniques presented a challenge to faithfully reproduce, we chose these techniques because they are heavily deployed in the field and require little optimization. The reviewer may choose to view the following publications (PMID: 37525760, 37525760, 38043608, 39191233, 39487193, 39633352, 39632860, 39611037, 39527321). Further, the presence of phosphor-Histone H3 in mitotic cells has been validated by pathologists in clinical samples (PMID: 25813470, 33007357, 231749360. The presence of both phosphor-Histone H3 and the >2N DNA content in ~10% of Hu+ cells provide concordant results on the presence of cell proliferation and mitosis in a subset of all Hu-expressing cells - and thus both these results taken together provide confirmation that ~10% of all Hu+ cells are mitotic cells and hence neuroblasts.

The authors can search publicly available open access databases and provide evidence for the presence of Histone H3 genes in enteric neurons. Such evidence would strengthen the paper.

We wish to remind the reviewer that the expression of Histone H3 does not indicate proliferating cells, but that it is the phosphorylation of this specific Histones (specifically H3) at a specific site Serine 10 that is accepted as a validated marker for proliferation. The antibodies that we used, specifically detect Histone H3 that is phosphorylated at Serine 10, and thus are well accepted as a specific marker for proliferating cells.

We also wish to direct the reviewer's attention to a recent study published in eLife (Kulkarni et al 2023), where using computational analyses that detects the expression of multiple cell cycle associated genes, we established the presence of cell proliferation in enteric neurons (in both neural crest and mesoderm-derived lineages) Regarding the immunohistochemistry. In the mouse, the authors use a very gentle fixation and processing and yet in the human the processing is much harsher. Very few people fix tissues for 5 min. So if the approach is to have the utility that is claimed, the authors should make it clear whether longer fixation times give comparable labeling, and/or the limitations of the approach. This would greatly enhance the value to the field and increase the importance of the paper.

We thank the reviewer for this comment. We use gentle fixation as a rule for isolated and then fixed LM-MP strips by following a recent report (Kulkarni et al, eLife 2023) that showed that a gentle fixation allows the immunolabeling of certain hard to immunostain antigens (such as MET) while preserving the ability to detect other antibodies. It is for this reason that we continue using gentle fixation times since we have observed the gentle fixation to be optimal for a wider range of antigens.

However, in response to their comment, we performed immunostaining of tissues fixed for longer time intervals and detected the presence of phosphor-Histone H3 immunostaining at longer fixation times. We have provided this new data in Figure 7 of the revised manuscript. However, Kulkarni et al 2023 shows that there is clearly a cut-off point where the tissues will be over-fixed for immunostainings not to work, and that these cut-off points may be different for different antigens and cells. Thus, to avoid these issues, we prefer to work with gentle fixation times.

There is strong evidence in support of nestin+ cells in the enteric nervous system that are proposed to be progenitor cells. In the model proposed it is suggested that some of the current mitotic neuroblasts are nestin+ cells. Double labeling to demonstrate this would have greatly strengthened this argument and is to my mind an essential step in the justification of the model proposed in Fig. 5.

While our resubmission has removed Figure 5 as a model figure, we agree with the reviewer that it is important to test the co-expression of Nestin in pH3 and Hu-immunolabeled cells.

To that, in the new Figure 5, we show the presence of a pH3-immunolabeled Nestin-expressing progenitor cell that does not express Hu. This is similar to an earlier report (Kulkarni, PNAS 2017) where the authors detected Ki67 in a subset (~2%) of Nestin-expressing progenitor cells. In addition, here we also provide evidence that pH3-immunolabeled Hu+ cells that are undergoing mitosis also express Nestin (Fig 6). This provides further evidence that in the adult myenteric ganglia, Nestin-expressing progenitor cells as well as their derivative Hu+ Nestin+ neuroblasts are both proliferating cells.

There is speculation regarding some of the cell types labelled. Addressing this with double labeling (e.g., glia could be labelled with S100 or Sox10) would remove that and strengthen the paper.

We thank the reviewer for their comment, but unfortunately due to technical limitation (i.e. lack of availability of a robust antibody against glial cells that is not made in rabbit), we were unable to test the nature of the other non-Nestin-expressing non-Hu-expressing cells in the myenteric ganglia.

Minor points.

The title and other places refer to enteric nervous system when the observations are restricted to the myenteric plexus. Please revise accordingly.

We thank the reviewer for this suggestion. We have amended the title and other references in the body of the manuscript.

Animals are "euthanized" not "sacrificed." We thank the reviewer for this proposed edit and have suitably changed the wording.

Reviewer 2 comments:

Since the percentage of cells exhibiting increased DNA content by FACS is substantial (>10% of total Hu+ cells) and if these cells are indeed "neuroblasts" progressing through the cell cycle they should be detectable as mitotic figures. The absence of such a finding raises the question of whether Hu is expressed in another cell type and introduces concerns about the interpretation of the results shown.

We thank the reviewer for this comment. To this, we first establish that the two Hu antibodies (commercial and ANNA1) detect the same ganglionic cells (Fig1). Next, we establish that that Hu-expression detects specifically PGP9.5+ cells (Fig 1, 2). These data, and the data generated by many other investigators who have routinely immunostained with Hu antibodies before, should provide confidence to the reviewer that both the commercially available (rabbit) antibody and the anti-Hu ANNA1 antiserum has never been reported to label non-neuronal structures in the LMMP layer.

Next, it was important for us to study to address this important comment and test whether the presence of phosphor-Histone H3 and presence of increased DNA content allowed us to find cells in the myenteric ganglia that appear binucleated or show nuclei that are in the process of nucleokinesis (dividing nuclei that appear as conjoined nuclei in a snap-shot of time). We are happy to report that we found instances of such Hu-immunolabeled cells in the adult small intestinal myenteric ganglia that are labeled both with antibodies against Hu and the other pan-neuronal marker we deployed: PGP9.5 (Fig 2). The orthogonal views of this image show the presence of two conjoined nuclei in the same Hu- and PGP9.5 immunostained cell (within dotted lines). We also observe other Hu-immunolabeled cells where the orthogonal views show the contiguity of Hu immunostaining (Fig 1-1, Fig 6), and one of this cell shows multiple nuclei - two of which are immunolabeled with antibodies against phosphor-Histone H3 (Fig 6).

To ensure that these observations are not due to issues with gentle fixation, we again observed the myenteric ganglia from tissues that were fixed for longer duration of time (Fig 7C, D) and found conjoined nuclei in Hu-immunolabeled cells, where one of the nuclei is labeled with antibodies against phosphor-Histone H3 and other nuclei are not. Parts of this cell are labeled with antibodies against Hu while another part is not, which is expected in asymmetric cell division.

We wish to remind the reviewer that cell division is a complex and diverse process, and cells may divide symmetrically (one parent cell dividing into two daughter cells) or asymmetrically (one parent cell retaining itself and budding to generate two or more asymmetric daughter cells). A review in Genes and Development provides an excellent resource on this topic (PMID: 19952104). Our data suggests that mitosis in the myenteric ganglia may occur through such mechanisms but elucidating the molecular and cellular biology of this process is an important challenge that should be interrogated by further analyses and studies. Studying these processes is beyond the scope of this study, as in this study, we are interrogating the presence of adult enteric neurogenesis in the healthy myenteric plexus using different tools to provide more confidence on the veracity of this observation.

Major Concerns:

1 - There are concerns regarding the FACS data presented: a. The authors need to provide a fully defined gating hierarchy that illustrates how they accomplished their gating and they need to clearly show that they have excluded doublet nuclei. Lack of pre-gating could lead to persistence of doublet nuclei in their sort profiles. b. There is a double hump for both the nucBlue and DAPI that is present in the region designated as the G2_M phase. Such a profile is quite unusual for this type of profile so it needs to be explained. c. There is a notable amount of signal that is off scale in the nucBlue plot (Figure 1C). What is the source of this? d. The placement of the right side border for the G2_M phase appears to be arbitrary. Why was this placement chosen? e. In Figure 1D, the placement of the S-phase gate appears to be arbitrary as it includes part of the primary peak indicated as G1phase nuclei. f. For Figure 1D, the presentation would be more convincing if DAPI versus side scatter were also shown.

We thank the reviewer for their comment on this Figure and especially on the flow analyses. This prompted us to seek expert opinion on how to best perform and depict FACS analyses.

To that we conferred with our FACS facility director to provide a better analysis of the existing data. In the revised figure and in the Fig 3 - 1, we are providing the requested gating hierarchies and DAPI vs side scatter gates for our analyses. In our conference, our FACS facility director informed us that it is not uncommon to find varying contours in the part of the bell curve, as no normal distribution is perfectly contoured in physiological assays. Our re-analyses of this data removed some of the debris that was off-scale and as a result the aberrant signals that were off-scale have now been removed. As a result of this, the proportions of G2/M phase cells have now changed and are now concordant between the assays performed at two different insitutions. We conferred again with our facility director on the placement of the S-phase gate and were informed that this typically includes all events between the G1 phase and the G2/M phase gates. In re-doing this analyses and figures, we have followed the advice of our facility director who has 15+ years of experience in flow sorting and analysis.

2 - Additional flow sort information is needed to demonstrate that all the Hu+ nuclei being analyzed are indeed neuronal. The authors should use a complementary pan-neuronal cell marker such as PGP9.5 or NeuN (being careful to use an antibody that has been validated in knockout tissues to ensure specificity) so they can confidently state that the FACS sorted nuclei with higher DNA content are indeed neuroblasts based on the co-localization of Hu with the additional pan-neuronal marker.

We thank the reviewer for their comment, but we humbly argue that Hu immunolabeling is specific to all myenteric cells that have been annotated as neurons. There are no other cell types that immunolabel strongly for Hu. In addition, at a recent conference on Enteric Nervous System held in Philadelphia, Hu-immunolabeling based flow sorting is the method used by multiple investigators. Further, a recent preprint use Elavl-specific transgenic mouse to label all enteric neurons (DOI https://doi.org/10.1101/2024.05.02.592229).

We also tested a few different antibodies to study whether they provide robust immunostaining of myenteric nuclei for FACS analyses. Unfortunately, these experiments failed either due to sub-optimal conjugation (since the antibodies were not directly conjugated), or due to poor signal. In our experience (and in the experience of other investigators), the signal quality of Hu antibodies (that are sold as directly conjugated) is unmatched. The Heuckeroth Lab had also previously tested whether NeuN could be used to sort out neuronal nuclei but similar to us, their endevour to use this antigen failed. See Table 1 in their report: https://pmc.ncbi.nlm.nih.gov/articles/PMC8099699/ 3 - Similarly, the authors should use a complementary pan-neuronal cell marker such as PGP9.5 or NeuN (being careful to use an antibody that has been validated in knockout tissues to ensure specificity) in immunohistochemistry (IHC) of mouse and human tissues to increase confidence that the Hu+, pH3+ cells they are detecting are neuronal in nature.

We thank the reviewer for their comment, and we have performed immunostaining with ANNA1 and PGP9.5 antibodies together (Fig 1) to show that Hu and PGP9.5 are expressed by the same cells.

4 - The authors state "we observed that >10% of these Hu+ nuclei show evidence of higher-than-expected DNA content, which indicates that these cells undergo DNA replication and mitosis". This finding indicates that the cells have a higher than 2N DNA content and suggests the possibility that DNA replication may have occurred. However, there are other mechanisms by which cells increase their DNA content and the authors have not considered these other possibilities or even mentioned them in their discussion. The authors could perform a short-term treatment of mice with inhibitors of DNA replication to determine if DNA replication is indeed occurring by comparison to mice only dosed with drug diluent. This is likely to be show a reduction of 2N nuclei by FACS or pH3+ cells by IHC given the frequency of dividing cells that is claimed in this study. Since the initial findings suggest that ~10-12% of cells have greater DNA content, reductions due to drug treatment should be readily detectable even if the treatment is for a short period of time.

We thank the reviewer for their comment and agree that there are many different methods through which cells cycle, and that not all mechanisms of DNA synthesis may lead to cell division. However, our revised manuscript provides evidence of multi-nucleated or bi-nucleated cells that 'together with' the pH3 (which only labels mitotic cells) and flow cytometric data (which shows Hu+ cells in S and G2/M phase), provide more evidence that the presence of higher-than-expected DNA content and the presence of pH3 is suggestive of cytokinesis. None of our current data or past reports suggests that processes whereby persistently high DNA content is maintained in cells are deployed in the myenteric ganglia at steady state. A recent paper showing the presence of Sox2 in ~10% of myenteric neurons in the adult healthy colon again provides the same inference.

In addition, commonly used mitotic inhibitors not only inhibit DNA synthesis or cytokinesis, but also promote apoptosis (for e.g. Cell Research volume 28, pages 544-555 (2018)). This would suggest that to perform an experiment as postulated by the reviewer would require us to titrate the dose of such drugs, and there is no clarity in the current literature as to what minimum dose would be optimal to test the effect of the drug selectively only on proliferation of myenteric cells (especially as alterations in allied immune cells such as macrophages would also have a significant impact on the biology of myenteric neurons).

We humbly argue with the reviewer that the presence of mitotic marker pH3 in ~10% of neurons, the presence of higher than 2N content of DNA in ~10% of neurons, the observation of bi-nucleated and multi-nucleated Hu-expressing cells in the adult healthy myenteric ganglia, the observation of ~10% Sox2+ cells (in a recent report in AJP-GI) in the adult healthy colonic myenteric ganglia, the presence of ~10% rate of apoptosis in healthy myenteric neurons as observed by us and others - all points to the same logical conclusion that ~10% of these Hu-immunolabeled cells are mitotic in nature and this rate is similar to the rate of apoptosis in the myenteric ganglia in healthy adult mice.

5 - The authors should scan tissues stained for Hu and pH3 for mitotic figures (cells that are in the process of dividing but have not yet undergone cytokinesis) and present evidence of this. There are cell membrane markers that could also be used to show that the duplicated nuclei are within the same cell membrane prior to cytokinesis.

We thank the reviewer for this comment, and to their comment, we have now provided additional marker PGP9.5 which is localized both in soma, nuclei, and fibers to observe cells that are in the process of dividing but not yet have divided (Fig 2).

Cell membrane markers or lipophilic dyes were found to label many different cell types both inside and outside of the ganglia and did not provide significant and sufficient contrast by which various cell types could be differentially observed, and thus we had low confidence on how to infer the data thus generated. By contrast, the immunolabeling with a validated intracellular pan-neuronal marker PGP9.5 provides adequate contrast between labeled and unlabeled cells to allow us to infer with confidence as to the contiguous nature of the Hu-immunolabeled cell.

6 - As indicated by the article title, the focus of this paper is on potential for proliferation of some precursor to form HuCD+ enteric neurons. The authors then add into Figure 2 a mention of cleaved caspase 3. This aspect is not necessary for the major focus of the article and is a bit distracting. The authors should remove the cleaved caspase aspects from Figures 2 and 3 so as not to distract the readers from their emphasis on neuroblast proliferation.

It has been previously posited that the presence of adult steady state neurogenesis can be understood only through the looking glass of neuronal homeostasis, whereby neurogenesis is a mechanism to offset the loss of apoptotic neurons and thus maintain the structure of the myenteric plexus. The presence of apoptotic neurons in the healthy myenteric plexus, which is more widely accepted than the observation of adult neurogenesis, is still debated in some quarters, and we retained this part of our manuscript to show that the observation of apoptosis (using Cleaved Caspase 3 specific antibodies) is robust and reproducible. We request the reviewer to allow us to keep this data in the manuscript.

7 - The authors present a final model in Figure 5 that refers to "NENs" and "MENs" that is not founded on the the data presented in this study. Moreover, the text states "we propose that populations of both neural crest- derived enteric neurons (NENs) and the mesoderm-derived enteric neurons (MENs) undergo steady state neurogenesis in adults." This statement appears to be a huge leap beyond the findings in this submission and there does not appear to be any basis for this statement in either reference 7 or reference 26. The authors need to scale back their conclusions and their model so they are consistent with the results presented.

We thank the reviewer for their comment, and we agree that it would be best to remove this from the current manuscript.

8 - Others have reported (Desmet et al., 2014 doi: 10.1111/nmo.12371) that Hu localization changes with hypoxia of tissues. This reviewer recommends that the authors focus on the proliferative aspects of this study and avoid the apoptosis aspect. However, they do need to keep in mind that the strong mostly nuclear label of Hu shown suggests that the tissues are hypoxic. This aspect may be contributing to the observed cleaved caspase 3 that is detected. If the authors perfuse the animals with fixative prior to dissection this complication could be avoided. This should be addressed as a possible explanation of the cleaved caspase 3 neurons that are seen if the authors aim to keep this aspect in the study.

It has been previously published in the rebuttal to authors commentary for a manuscript on Biorxiv (Rebuttal... in https://www.biorxiv.org/content/10.1101/2020.08.25.262832v6.supplementary-material) that the Desmet et al paper used an antibody against Hu (generated in mouse) and observes increasing nuclear colocalization of Hu signal in response to tissues remaining unfixed post-isolation for more than 1 hour. This is not the antibody used by us in our analyses, and our protocols allow for a quick isolation and fixation of tissues that take less than an hour and do not change tissue structure or cell types. Further, even in the Desmet et al paper, the authors observed 5 - 10% of cells with nuclear co-localization of Hu even immediately after isolation (see reproduced figure from their article). This suggests that the nuclear co-localization of Hu (which does not change significantly in the first hour - a time far longer than taken by us for our analyses) does not change as a result of hypoxia in the first hour. There is currently no data that links the nuclear co-localization of Hu at steady state (immediately after isolation) or within the first hour to increased cleaved caspase 3 immunoreactivity, and hence it would be a leap for us to address this as a possible explanation for Cleaved Caspase 3 (CC3) immunoreactivity. CC3 immunoreactivity is observed by many labs that have used varying protocols for fixation, and the congruency of all their data suggests that the continual rate of apoptosis is a process that occurs at steady state in the healthy adult gut.

Minor Points 9 - the authors should make sure that all figure labels are larger and clearly legible as there are multiple aspects that are too small for figure production (e.g. labels of phases and axes in Figure 1; Figure 2 panel labels).

We thank the reviewers for the suggestion and have tried our best to make the fonts legible. In certain cases, the file sizes are large and the conversion to smaller sizes may impact the legibility and unfortunately these issues are beyond our control.

10 - The first results section subheading is "Adult murine small intestinal myenteric neurons show evidence of mitosis." However, what the authors show is increased amounts of DNA in a small percentage of Hu+ nuclei. They do not show evidence of neuronal cells undergoing mitosis (cell division) in this section. The subheading should be revised so it is not over-stating the results.

We thank the reviewer for this suggestion and as a result, we have revised the manuscript with new data and have taken measures to not over-interpret our findings 11 - The authors should provide additional details about their methods so that others may repeat their analysis: a - Vendor name and catalog number should be provided for all reagents including wide-bore, low retention 1000 μl pipette tips and cell sieves We have provided more information in the methods to help address this issue. b - The authors have been thorough in listing information for antibodies however they should also include RRIDs for the antibodies they have used.

We have now provided the RRIDs for all antibodies used in this study.

C - The source of ANNA-1 antibody is not listed and should be included. Since this appears to be a patient antibody is this use properly permissioned? We have provided more information in the methods to help address this issue. The ANNA1 antisera was procured from Dr. Sean Pittock at Mayo Clinic who procured it from Dr. Vanda Lennon after she retired.

12 - It is not clear why the authors use the direct-labeled antibody for FACS and then use the ANNA-1 antibody for staining intact tissues. This difference should be explained since antibodies from different sources can recognize different epitopes.

Directly conjugated antibodies work best for FACS analyses as the conjugation has been optimized by the vendor and thus, can be used directly on delivery. On the other hand, ANNA1 allows us to use commercial antibodies generated in rabbit for co-immunolabeling (for example against CC3 or pH3) and conjugation of ANNA1 antibodies from human serum did not work well and would require further optimization. Fig 1 shows that ANNA1 and the commercial antibodies both detect the same cells and cellular structure suggesting that they do not differ significantly in their detection of neurons.

13 - The citation for reference #14 appears to be incomplete.

We have edited this reference and have check it to ensure it is complete.
