# Supplementary figures and images for: Detection of Mitotic Neuroblasts Provides Additional Evidence of Steady-State Neurogenesis in the Adult Small Intestinal Myenteric Plexus
Source: eNeuro. 2025 Mar 5;12(3):ENEURO.0005-24.2025. doi: 10.1523/ENEURO.0005-24.2025 (PMC11884873; doi:10.1523/ENEURO.0005-24.2025)

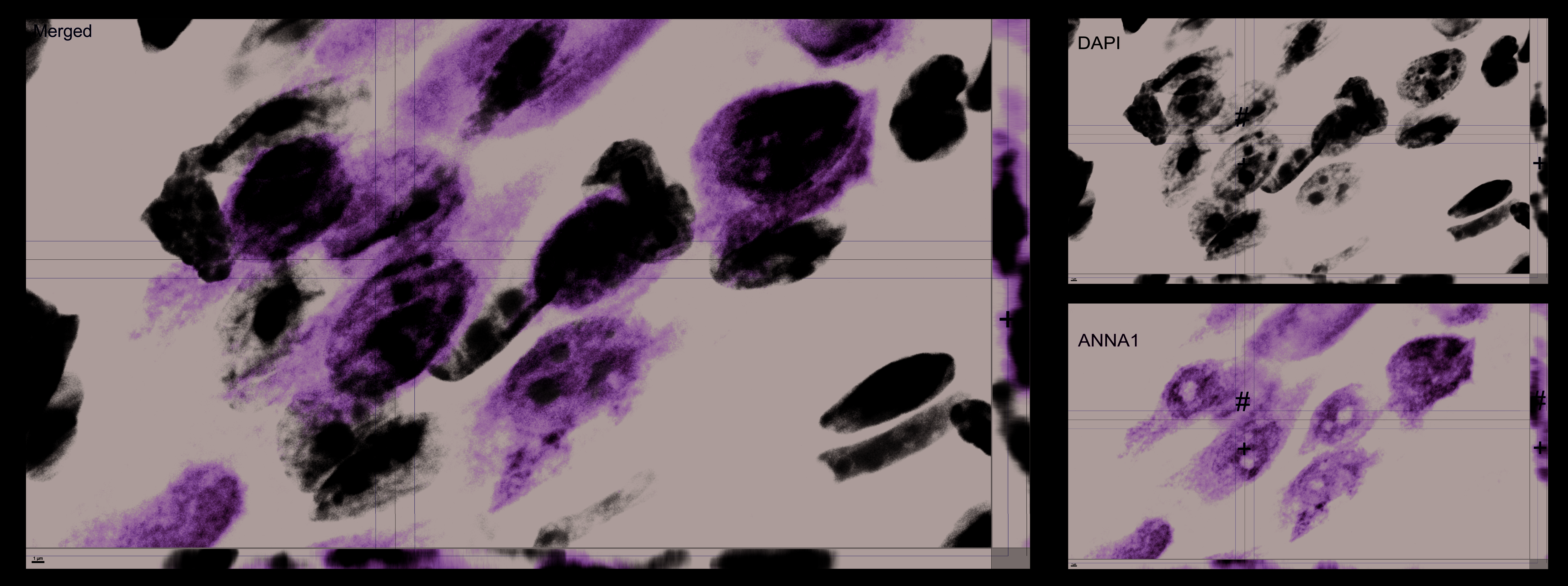

Supplement: Figure 2-1 — Observation of bi-nucleated or conjoined nuclei in Hu+ myenteric cells. Orthogonal color-merged and color-segregated views of an image of a myenteric ganglion from an adult murine small intestinal tissue, where the tissue is immunolabeled with antibodies against Hu (green) and stained with nuclear dye DAPI (grey) shows the presence of two near or conjoined nuclei # and + in a contiguous Hu-immunolabeled cell. Scale bar denotes 1 µm. Download Figure 2-1, TIF file. [file eneuro-12-ENEURO.0005-24.2025-s002.tif]

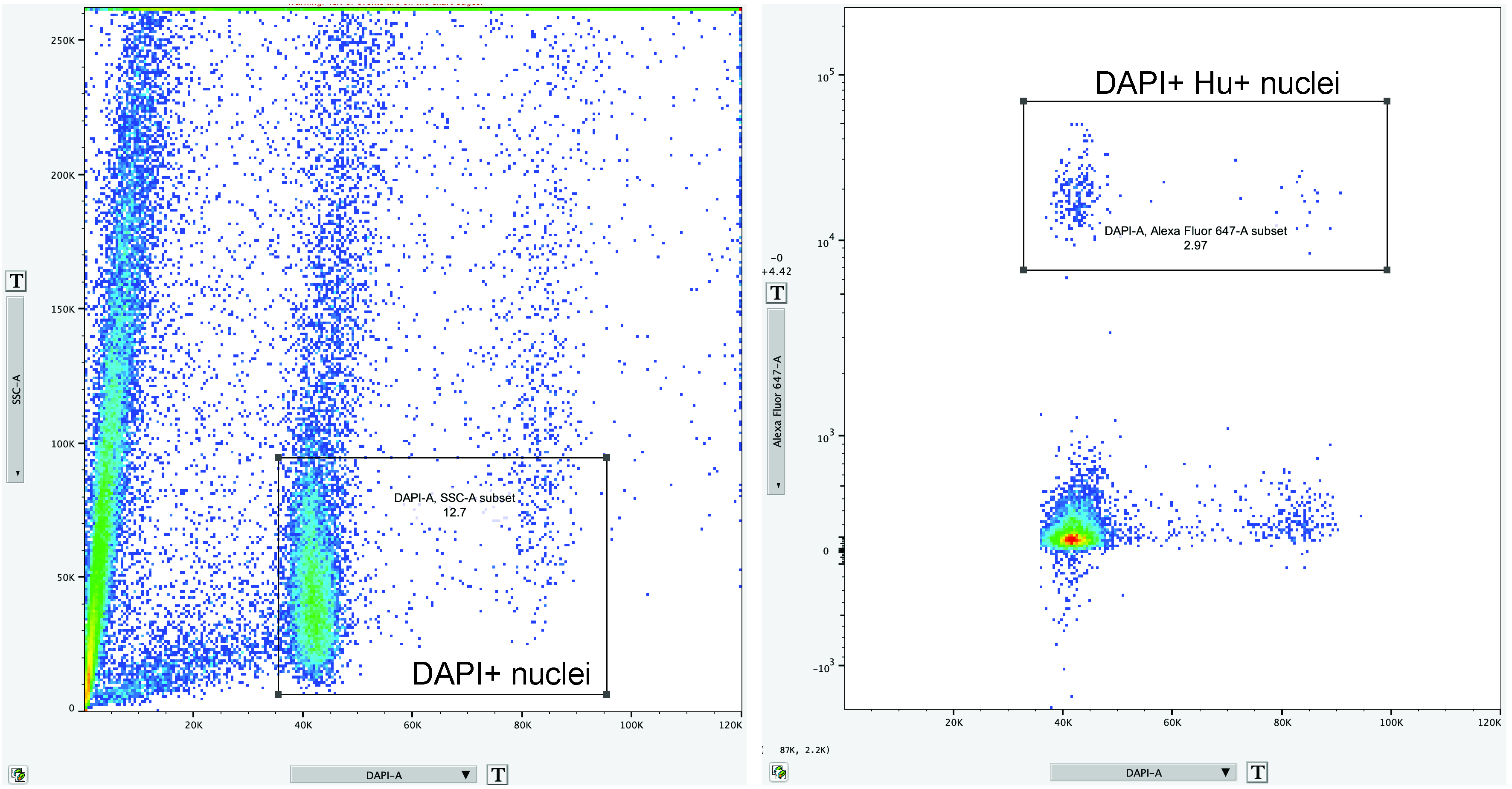

Supplement: Figure 3-1 — Flow gates used for assessing Hu-immunolabeled nuclei from adult small intestinal LM-MP tissues from mice in the Johns Hopkins colony. Fixed nuclei isolated from adult murine small intestinal LM-MP were stained with nuclear dye DAPI and directly conjugated Hu antibody (Alexa 647) and assessed to establish the gates for DAPI + nuclei (left plot), and DAPI + nuclei that immunolabeled for Hu (right plot). Download Figure 3-1, TIF file. [file eneuro-12-ENEURO.0005-24.2025-s003.tif]
